# Transcriptomic Response of Resistant (PI613981–*Malus sieversii*) and Susceptible (“Royal Gala”) Genotypes of Apple to Blue Mold (*Penicillium expansum*) Infection

**DOI:** 10.3389/fpls.2017.01981

**Published:** 2017-11-16

**Authors:** Ana-Rosa Ballester, John Norelli, Erik Burchard, Ahmed Abdelfattah, Elena Levin, Luis González-Candelas, Samir Droby, Michael Wisniewski

**Affiliations:** ^1^Instituto de Agroquímica y Tecnología de Alimentos (CSIC), Valencia, Spain; ^2^United States Department of Agriculture–Agricultural Research Service, Kearneysville, WV, United States; ^3^Dipartimento di Agraria, Università Mediterranea di Reggio Calabria, Reggio Calabria, Italy; ^4^Agricultural Research Organization, Volcani Center, Bet Dagan, Israel

**Keywords:** *malus domestica*, *Penicillium expansum*, qualitative trait loci, postharvest pathogens, wound healing, necrotrophic pathogens

## Abstract

*Malus sieversii* from Central Asia is a progenitor of the modern domesticated apple (*Malus* × *domestica*). Several accessions of *M. sieversii* are highly resistant to the postharvest pathogen *Penicillium expansum*. A previous study identified the qM–*Pe3*.1 QTL on LG3 for resistance to *P. expansum* in the mapping population GMAL4593, developed using the resistant accession, *M. sieversii* –PI613981, and the susceptible cultivar “Royal Gala” (RG) (*M. domestica*), as parents. The goal of the present study was to characterize the transcriptomic response of susceptible RG and resistant PI613981 apple fruit to wounding and inoculation with *P. expansum* using RNA–Seq. Transcriptomic analyses 0–48 h post inoculation suggest a higher basal level of resistance and a more rapid and intense defense response to wounding and wounding plus inoculation with *P. expansum* in *M. sieversii* –PI613981 than in RG. Functional analysis showed that ethylene–related genes and genes involved in “jasmonate” and “MYB–domain transcription factor family” were over–represented in the resistant genotype. It is suggested that the more rapid response in the resistant genotype (*Malus sieversii–*PI613981) plays a major role in the resistance response. At least twenty DEGs were mapped to the qM–*Pe*3.1 QTL (*M* × *d* v.1: 26,848,396–28,424,055) on LG3, and represent potential candidate genes responsible for the observed resistance QTL in *M. sieversii*–PI613981. RT–qPCR of several of these genes was used to validate the RNA–Seq data and to confirm their higher expression in MS0.

## Introduction

Worldwide, blue mold of apples, caused by *Penicillium expansum* Link, is regarded as one of the most important postharvest rots of apple fruit (Capellini et al., 1987; Jurick et al., 2011). *P. expansum* is also of great concern to fruit processing industries (juicing, baby food, ready to eat salads) due to its production of patulin, a mycotoxin which can contaminate infected produce and its products (Wouters and Speijers, 1996). The pathogenicity of *P. expansum* is not limited to apples but rather it has a wide host range, that includes pears, peaches, nectarines, plums, and apricots (Sanzani et al., 2013). The disease cycle of blue mold is well known. Since *Penicillium* is unable to penetrate its host tissue directly, it can only infect fruit through wounds that usually develop during harvesting and packaging. The sexual stage of this pathogen has not been identified and its dissemination relies on dispersal by asexual spores (conidia). *Penicillium* spores are ubiquitous in orchards, packinghouses, and storage facilities. The spores that eventually occupy fruit wounds, germinate and grow rapidly, visibly macerating the tissue within 48 h. A unique feature of *P. expansum* compared to other postharvest pathogens, is its ability to grow at low temperatures. This ability allows it to survive and grow during fruit low–temperature storage. Blue mold decay is especially a problem in production systems that do not use pre- or postharvest fungicides (Tahir et al., 2015).

While apple producers are reliant upon the use of synthetic fungicides for decay control, the future postharvest use of fungicides is in question due to the severe restrictions or complete bans instituted within the European Community (Droby et al., 2016b; Wisniewski et al., 2016). Alternative postharvest strategies, based on biological (microbial) and other physical and food grade substances (natural plant and animal products), are being pursued (Droby et al., 2016a; Wisniewski et al., 2016). The use of these alternatives, however, remains very limited due to their reduced efficacy and/or inconsistent performance, relative to synthetic fungicides, under commercial conditions.

One of the most effective and safest means to control plant diseases has been the use of resistant cultivars. Janisiewicz et al. (2008) and Jurick et al. (2011) have both noted a lack of resistance to *P. expansum* (blue mold) in commercial apple germplasm. In this regard, a set of 81 apple cultivars adapted to cool climates (Norway and Sweden) were screened for resistance to blue mold during low-temperature storage for 6 or 12 weeks, depending on whether the cultivar was early-or late-maturing (Tahir et al., 2015). While distinct differences in susceptibility to *P. expansum* were noted in their study, the level of resistance was associated with firmness and the rate of softening in storage. More explicitly, cultivars with firm apples were more resistant to blue mold than were apples that softened slowly in storage. Thus, it appears that ripening characteristics are indirectly the source of blue mold resistance rather than any direct form of genetic resistance. Association between firmness and resistance/susceptibility to blue mold has also been noted in other studies (Vilanova et al., 2012; Ahmadi-Afzadi et al., 2013). However, little attention has been devoted to postharvest disease resistance in apple breeding programs (Janick and Moore, 1996). This is due both to a lack of sources of genetic resistance and the current prohibitive cost of maintaining trees in trial plots for several years. Therefore, the identification of a genetic source of resistance to blue mold as a heritable trait, and not directly linked to firmness, would represent a significant accomplishment and the identification of DNA markers for resistance to postharvest decay would also increase the feasibility of breeding resistant cultivars through marker-assisted selection (MAS) of seedlings prior to field planting.

USDA-sponsored expeditions to Central Asia have allowed the establishment of a large collection of *Malus sieversii* accessions, which are maintained at the USDA Plant Genetics Resources Unit (PGRU) in Geneva, NY (Hokanson et al., 1997; Luby et al., 2001; Forsline and Aldwinckle, 2004). In contrast to other wild *Malus* species, *M. sieversii* is recognized as an excellent source of disease resistance for scion breeding because of the unique occurrence of larger and more palatable fruit within the species. Seven elite lines of *M. sieversii*, selected for resistance to apple scab were crossed with RG and segregating field-grown populations were established. PI 613981, the *M. sieversii* parent used to establish the GMAL4593 mapping population, is resistant to blue mold (Janisiewicz et al., 2008). This population was genotyped and phenotyped for postharvest resistance to *P. expansum* over a four–year period with the objective of identifying heritable genetic markers for blue mold resistance. Norelli et al. (2017) identified a quantitative trait locus (QTL) on LG3 of apple, qM–*Pe3.1*, that accounts for almost 30% of the observed variation in blue mold resistance in the GMAL4593 mapping population, and determined that resistance at this locus was due to an allele from *M. sieversii* (PI613981). Another significant QTL for blue mold resistance, qM-*Pe*10.1, was identified on LG10 where many ripening-related genes are located (Costa et al., 2010). An allele from RG appeared to be the main contributor to resistance at this QTL but resistance alleles from both parents appear to act additively at the QTL located on LG10. The resistance on LG10 was most likely related to the ripening–related resistance noted by Vilanova et al. (2012) and not the active resistance response noted by Janisiewicz et al. (2008). Full details on the genetic basis of resistance are discussed in Norelli et al. (2017).

In the past two decades, the use of transcriptomic and proteomic analyses of plants, in relation to growth, and development, stress-adaptation, and disease resistance, has increased exponentially (Tian et al., 2016; Wisniewski et al., 2016; Simsek et al., 2017). These approaches have become more readily available as the number of sequenced plant genomes has increased and sequencing technology has improved and become more cost-efficient. Microarray-based studies of resistance to *P. expansum* in apples have noted the involvement of several defense-related genes in the resistance response, as well genes involved in detoxifying reactive oxygen species (ROS). These findings were revealed by comparing the response of apple fruit to a pathogen (*P. expansum*) and a non-pathogen (*P. digitatum*) (Vilanova et al., 2014) or by comparing the presence of polyphenolic compounds in “resistant” and “susceptible” commercial cultivars of apple (Ahmadi-Afzadi et al., 2015). The role of the oxi-proteome in response to wounding and infection by *P. expansum* or *P. digitatum* was further documented by Buron-Moles et al. (2015). The majority of biochemical studies related to *P. expansum* pathogenicity have focused on extracellular cell-wall degrading enzymes, such as pectate lyases, and polygalacturonases (Wattad et al., 1994; Prusky et al., 2004; Prusky and Lichter, 2008). Pathogen modulation of the pH in the infection court, resulting in optimal conditions for the production and activity of these enzymes has been also reported (Prusky et al., 2004).

The present study was conducted to determine differences in gene expression in harvested apples of RG (susceptible) and *M. sieversii*–PI613981 (resistant) in response to wounding and inoculation with *P. expansum*. These two genotypes served as the parents of the GMAL4593 mapping population, which was used to identify QTLs for resistance to *P. expansum* (Norelli et al., 2017). RNA-Seq was used to focus on the early response (0–48 h post-inoculation) of these genotypes and identify differentially expressed genes (DEGs). Principal Coordinate Analysis (PCoA) was used to cluster the DEGs, while GO terms and MapMan BINs were utilized to provide insight into gene function. Special consideration was given to identifying DEGs located within the qM-*Pe3.1* QTL on LG3 for resistance to *P. expansum*.

## Materials and methods

### Fruit treatment

Mature fruit from *M. sieversii*–PI613981 (MS) and *Malus* × *domestica* RG were collected from trees located at the USDA–ARS, PGRU, Geneva, NY, on August 21, 2013 and shipped overnight to the USDA-ARS, Appalachian Fruit Research Station, Kearneysville, WV. All fruit were stored at 2°C until used in the experiments. Fruit were left in the lab overnight to come to room temperature before the experiment was started. Prior to wounding, a quality assessment of starch, firmness, weight, soluble solids, and titratable acidity was conducted on a subset of fruit as described in Norelli et al. (2017). Prior to wounding and subsequent inoculation, selected fruit were surface sterilized by dipping for 1 min in 2% bleach solution, rinsed with water, and allowed to dry and equilibrate to 20°C in plastic containers lined with fiber-based packing trays. Biological replicates were defined as groups of five apples, and each sample consisted of three biological replicates for each genotype. Control samples (T0) consisted of collecting peel and flesh tissue from unwounded, non-inoculated fruit at the start of the experiment. Fruit samples were subjected to the following treatments: wounded fruit, mock-inoculated with 20 μL of sterile water (denoted as W), or wounded fruit inoculated with 20 μL of a 1 × 10^4^ spores mL^−1^ suspension of *P. expansum*, strain PE 100 (denoted as P). Strain PE 100 is an aggressive strain of *P. expansum* (Janisiewicz et al., 1992; Ballester et al., 2015). Wounds were administered with a nail to a depth and diameter of 8 mm using a self-made wounding device. For wounded and *P. expansum*-inoculated treatments, 4 wounds, equidistant from each other, were made just above and around the equator of the fruit. Sampling was performed by removing 8 mm diameter plugs of tissue with a cork-borer, centered on the wound site, which were then immediately sectioned into small disks with a razor blade and flash–frozen in liquid nitrogen for storage at −80°C. Thus, each biological replicate contained 20 discs (5 fruits and 4 wounds per fruit). The removed plugs of tissue included peel tissues surrounding the wound and 4 mm of mesocarp (flesh) tissue. Fruit samples were collected at 0, 6, 24, and 48 h post inoculation (hpi). Storage temperature over the course of the experiment was maintained at 20°C. All fruit was kept in a closed plastic tray. A high relative humidity was maintained in each tray using wet paper towels that were rehydrated every 24 h.

### RNA extraction, library preparation, sequencing, and read processing

Lyophilized sampled tissue plugs were ground in liquid nitrogen. RNA extraction was performed using a slightly modified CTAB buffer-based protocol in which Qiagen RNeasy columns were used in place of more typical precipitation methods to capture RNA from the aqueous phase following phase-separation with chloroform (Mellidou et al., 2014). Each RNA sample was adjusted to contain 5 μg of total RNA. Library construction was performed using the protocol outlined in Zhong et al. (2011) and run in two lanes using the Illumina HiSeq2000 platform to obtain 51-bp single-end reads. Three independent biological replicates per sample were analyzed. Reads were deposited in the NCBI Sequence Read Archive (SRA) accession ID SRP105163 (BioProject ID PRJNA383305).

### Bioinformatics analyses

Adapter trimming, removal of low-quality reads, and subsequent processing of high-quality reads for each sample were conducted using the RNA-Seq analysis programs within the CLC Genomics Workbench v 8.0.2 (Qiagen, Valencia, CA USA). High-quality reads were obtained by removing low-quality bases with a Phred score lower than 13 (base-calling error probability limit = 0.05) and low-quality reads with ambiguous nucleotides. For gene expression analysis, RNA-Seq reads for each treatment were mapped to the *Malus* × *domestica* whole genome v1.0 (Velasco et al., 2010) using the default conditions established in the CLC Genomics Workbench pipeline for mapping reads with multiple hits to the reference genome:: mismatch cost = 1, insertion cost = 3, deletion cost = 3, length fraction = 0.5, similarity fraction = 0.8, maximum number of hits for a read = 10. When a read matched equally well to more than one place in the genome, it was randomly assigned to one of these places following an estimation algorithm, unless there were more than 10 possible places, in which case the read not was not mapped.

Quantification of transcript abundance and identification of differentially expressed genes (DEGs) were based on normalized gene expression values calculated as reads per kilobase of exon model per million mapped reads (RPKM) (Mortazavi et al., 2008). Constitutive differences between both parental lines were identified by analysis of DEGs between RG and MS at time 0 with the Baggerley's test as implemented in CLC Genomics Workbench based on a false discovery rate (FDR), corrected *p* ≤ 0.01. This test compares the proportion of counts in a group of replicates against those of another group of replicates, comprising weighted *t*-type test statistics (Baggerly et al., 2003). In order to compare the differences in gene expression in both parental lines in response to *P. expansum* infection, we normalized each RPKM value with the RPKM value at time 0 for the corresponding genotype (e.g., MS6P/MS0 and RG6P/RG0). Then, a two–factor ANOVA (FDR *p* ≤ 0.01) using the software MeV v4.9.0 (MultiExperiment Viewer) (Howe et al., 2011) was utilized in order to identify the interactions between genotypes and infection time course. Also, a *t*-test analysis (FDR *p* ≤ 0.01) was used to identify DEGs between both parental lines [i.e., log_2_(MS6P/MS0) vs. log_2_(RG6P/RG0)] at each time point. Lastly, one-factor ANOVA was used to obtain the DEGs in infected MS fruit [ANOVA of log_2_(MS6P/MS0 vs. log_2_(MS24P/MS0) vs. log_2_(MS48P/MS0); FDR *p* ≤ 0.01) and in infected RG fruit (ANOVA of log_2_(RG6P/RG0 vs. log_2_(RG24P/RG0) vs. log_2_(RG48P/RGS0); FDR *p* ≤ 0.01] during the time course of the infection. Multivariate analysis, such as Principal Coordinates Analysis (PCoA) and construction of heatmaps, were performed using Qlucore v3.2 (Qlucore, Lund, Sweden) bioinformatic software. InteractiVenn (http://www.interactivenn.net/) online software was used to construct the Venn diagrams (Heberle et al., 2015). Analysis of biological significance was based on gene ontologies (GOs) using Singular Enrichment Analysis (SEA) and Parametric Analysis of Gene Set Enrichment (PAGE), both available at the AgriGO web site (http://systemsbiology.cau.edu.cn/agriGOv2/index.php) (Tian et al., 2017). DEGs were classified into MapMan BINs using the *Malus* × *domestica* genome, available in Phytozome v9.0, and their annotated functions were visualized using the MapMan tool (http://mapman.gabipd.org/) (Thimm et al., 2004).

### RT-qPCR analysis

In order to validate the RNA-Seq analysis, reverse transcription-quantitative polymerase chain reaction (RT-qPCR) was performed as previously described (Wisniewski et al., 2015). Total RNA was diluted to 12.5 ng μL^−1^. RT-qPCR analysis was performed using the Invitrogen SuperScript III Platinum SYBR Green One-Step RT-qPCR Kit with ROX (ThermoFisher Scientific, Waltham, MA, USA), with each reaction containing 25 ng of input RNA and 2 pmol of each primer; no-RT control reactions were included to ensure no residual DNA contamination. The Applied Biosystems ViiA 7 (ThermoFisher Scientific, Waltham, MA, USA) was set to cycle as follows: cDNA synthesis at 48.0°C for 30 min; 95.0°C denaturation for 5 min; 40 cycles of 95.0°C for 15 s followed by 55°C annealing for 1 min; followed by the default ViiA 7 hold and melt curve stages. Gene-specific primers were designed using CLC Genomics Workbench (Qiagen, Valencia, CA, USA) (Supplementary Table 1). Primers were verified for specificity by using genomic DNA templates and assessing the resulting amplicon by agarose gel electrophoresis and by RT–qPCR with a subset of the sample RNA on the ViiA7. All primers produced a single band and single peak. Primer efficiency was also verified for all primer sets by RT-qPCR analysis of a standard curve constructed by serially diluting RNAs from the sample set starting at some concentration above what was used in unknown samples and ending at a concentration well below it. Three technical replicates were used for each of three biological replicates. The *FYPP3* gene along with other endogenous reference genes (*LTL1, translation elongation factor 2, CKB4*, and *26S rRNA*) were assessed as to the stability of their expression within the two genotypes and across time points (Bowen et al., 2014). *FYPP3* was deemed the best overall reference gene using NormFinder software (Andersen et al., 2004). Expression levels of each of the analyzed genes were calculated using the comparative Ct (threshold cycle) method. Data from biological replicates were used to calculate mean ± standard error (SE) expression values.

## Results

### RNA-Seq transcriptome profiles

Apples from MS and RG trees were used to conduct a RNA-Seq analysis in order to characterize the expression profiles of apple genes in healthy, non-wounded fruits at the onset of the experiment (T0), and then in subsequently wounded fruit (W), or wounded and *P. expansum*-inoculated fruits (P) over a 48 h time course. Fruits of both MS and RG were also evaluated for different quality parameters (weight, firmness, starch, etc.,). While fruit from the MS parent were generally smaller than RG fruit, both had similar starch levels and firmness at the time of use (Supplementary Table 2). A subset of apples from the two genotypes were wounded and inoculated with 20 μL of a 1 × 10^4^/mL spore suspension of *P. expansum*, isolate PE 100 (PEX2), and stored at 20°C for 7 days to illustrate the resistant and susceptible response of the two parents (Supplementary Figure 1). Average lesion length was 0.00 mm and 13.82 ± 5.71 mm in MS and RG, respectively. Other subsets of fruit were collected for RNA-Seq analysis at 0, 6, 24, and 48 h post inoculation (hpi).

The high-throughput sequencing resulted in a total of 129.36 million, high-quality, single-end reads for MS (87.1% of the raw reads) and 137.18 million for RG (85.6% of raw reads) from time 0 untreated, and 6, 24, and 48 h W and P samples combined. These were designated as clean reads (Table 1 and check Supplementary Table 3 for each sample analyzed). An average of 85.5 and 92.1% of the clean reads for MS and RG samples, respectively, were successfully mapped to the apple reference genome v1.0 (Velasco et al., 2010). We have used not very restrictive mapping parameters in order to facilitate the mapping of *M. sieversii* reads against the reference genome (*Malus* x *domestica*), two different but very closely related species. Further analysis of the combined RNA-Seq reads that aligned to the apple genome resulted in the identification of an average of 52,493 and 53,408 protein-coding transcripts for MS and RG, respectively. This represents 91.5 and 93.1%, respectively, of the total predicted transcripts in the apple reference genome v1.0.

**Table 1:** Mapping characteristics of *Malus sieversii* PI613981 (MS) and “Royal Gala” (RG) parental lines in all samples analyzed (healthy tissue at time 0, wounded and wounded–inoculated tissues at 6, 24, and 48 hpi) to the reference genome *Malus* × *domestica* v1.0 (see Supplementary Table 3 for detailed information for each library, each replicate).

**Sample**	**Raw reads**	**Clean reads**	**Reads mapped onto the *M. domestica* v1.0**	**Number of Identified Genes**
**MS**				
Sum	148,488,797	129,356,853 (87.1%)	110,607,140 (85.5%)	
Average Reads per Sample	7,070,895	6,159,850	5,267,007	52,493
**RG**				
Sum	160,199,881	137,184,823 (85.6%)	126,281,310 (92.1%)	
Average Reads per Sample	7,628,566	6,532,611	6,013,396	53,408

**Figure 1:**
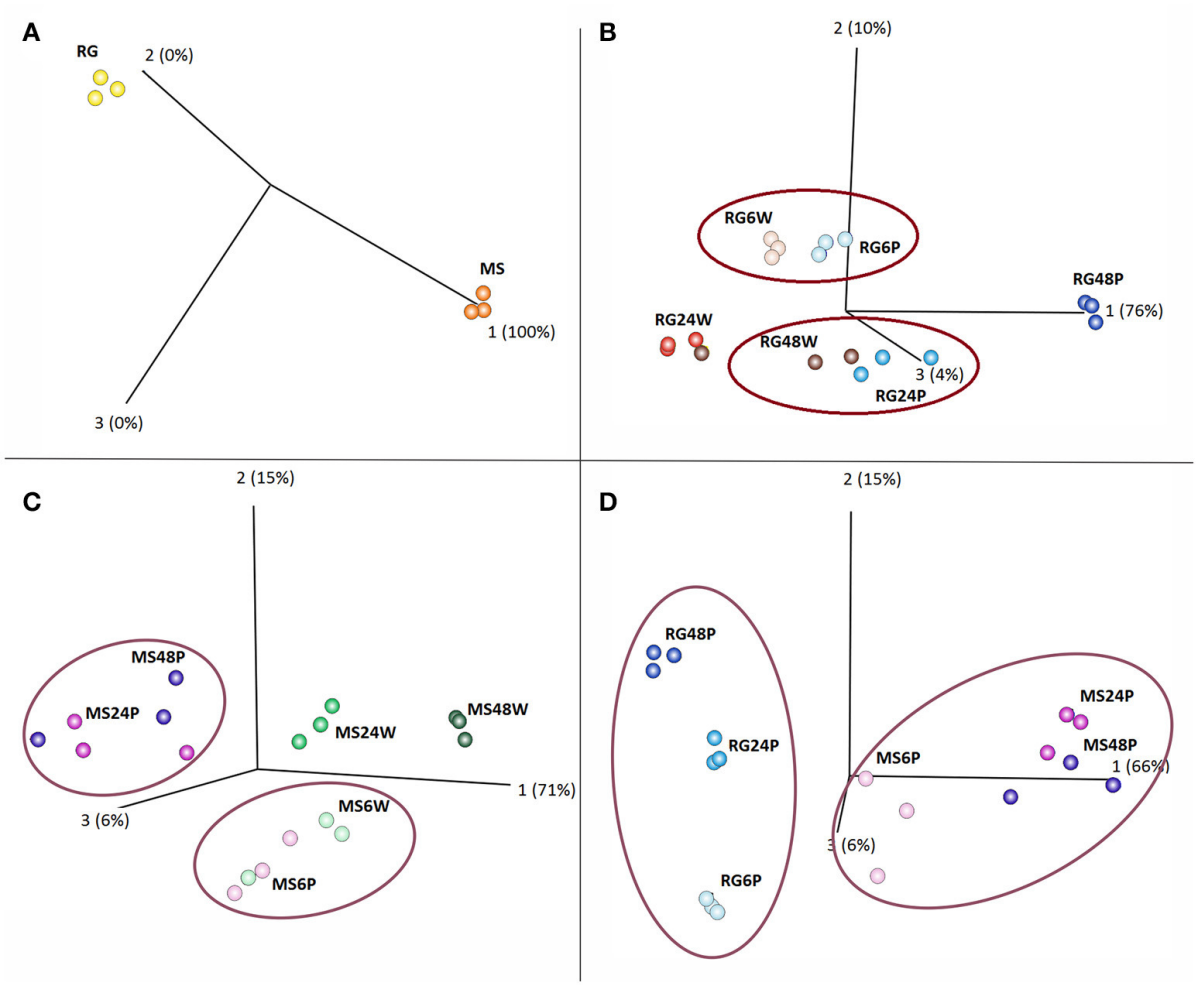
Principal Coordinates Analysis (PCoA) of *Malus sieversii*–PI613981 (denoted as MS) vs. “Royal Gala” (denoted as RG) parental lines. **(A)** PCoA plot at time 0; **(B)** PCoA plot of wounded (denoted as W) vs. *Penicillium expansum* inoculated (denoted as P) RG apple fruits at 6, 24, and 48 h; **(C)** PCoA plot of W vs. P MS apple fruits at 6, 24, and 48 h; and, **(D)** PCoA plot of *P. expansum* inoculated (P) RG vs. MS apple fruits at 6, 24, and 48 hpi.

PCoA of various combinations of the data revealed that the samples clustered into several distinct groups. As illustrated in Figure 1A, MS and RG clearly clustered into two distinct groups, even at time 0 (prior to W and P). PCoA and hierarchical clustering comparing gene expression in wounded (W) and inoculated (P) RG fruit indicated that gene expression over the time course of sampling (6, 24, and 48 hpi) was different in RG apples inoculated with *P. expansum* than in RG apples that were just wounded (Figure 1B and Supplementary Figure 2). Results of the PCoA analysis of RG apples indicated that a specific response to *P. expansum* did not occur until at least 24 hpi since samples of W and P fruit clustered together at 6 hpi. Furthermore, at 24 hpi, wounded/inoculated (P) samples were similar to 48 hpi samples of wounded RG fruit, while 48 hpi samples of wounded/inoculated fruit formed a unique cluster distant from all of the other clusters.

PCoA and hierarchical clustering of MS W vs. P samples over the time course of the experiment (6, 24, and 48 hpi) also revealed distinct clustering (Figure 1C and Supplementary Figure 3). In contrast to the RG fruit, data indicate that a specific response to inoculation occurred somewhere between 6 and 24 hpi in MS fruit and that 24 and 48 hpi wounded/inoculated samples (MS24P and MS48P) clustered in a group very distinct from the wounded 24 and 48 hpi samples (MS24W and MS48W). Furthermore, PCoA comparing wound response in the two genotypes revealed that each genotype clustered into separate distinct groups (Supplementary Figure 4). Time points within each genotype were largely distinct from each other, except for the MS 24 and 48 h samples which exhibited some proximity to each other. Lastly, the PCoA and the hierarchical clustering of P samples of the two genotypes also revealed that the two genotypes clustered independently of each other and that distinct groupings and clusters within each genotype were present related to the time of sampling (Figure 1D and Supplementary Figure 5). Even more evident in the clustering of the P samples (Figure 1D) than in the W samples (Supplementary Figure 4) was the close grouping of the 24 and 48 hpi MS clusters. In contrast, the 24 and 48 hpi RG samples clustered into distinct groups. The list of genes grouped together in MS24P and MS48P in the PCoA analysis is enriched in oxidative stress and PR–encoding genes.

### Differential gene expression in the RG and MS parental lines

**Figure 2:**
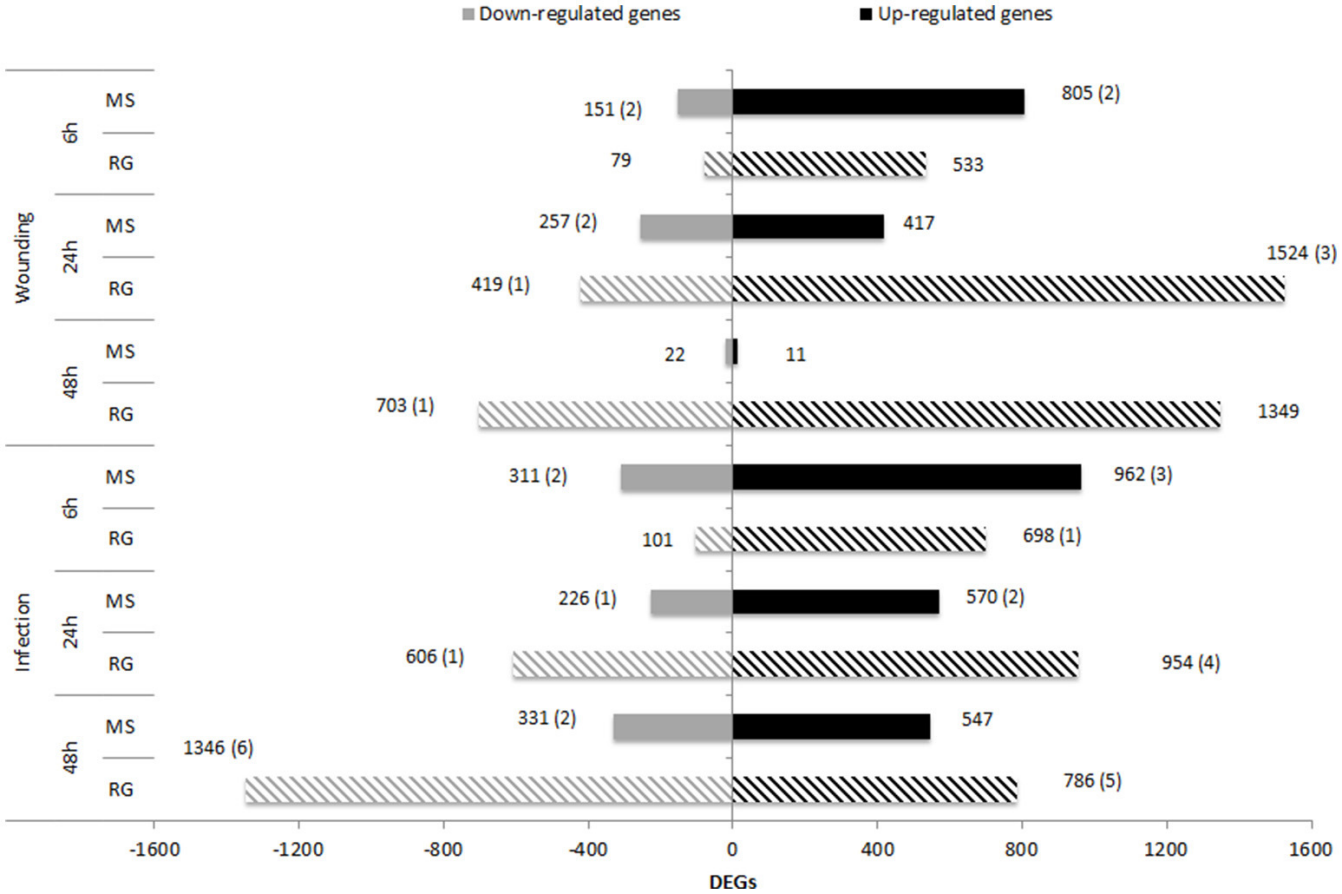
Number of up– and down–regulated genes [FDR *p* ≤ 0.01 and log_2_(ratio)≤-1 or log_2_(ratio)≥1] in MS (solid bars) and RG (striped bars) parental lines at 6, 24, and 48 h after wounding or after *P. expansum* inoculation compared to non–treated apples (Time 0). Numbers between parentheses indicate the number of DEGs located within the qM–*Pe3*.1 QTL on LG3.

**Figure 3:**
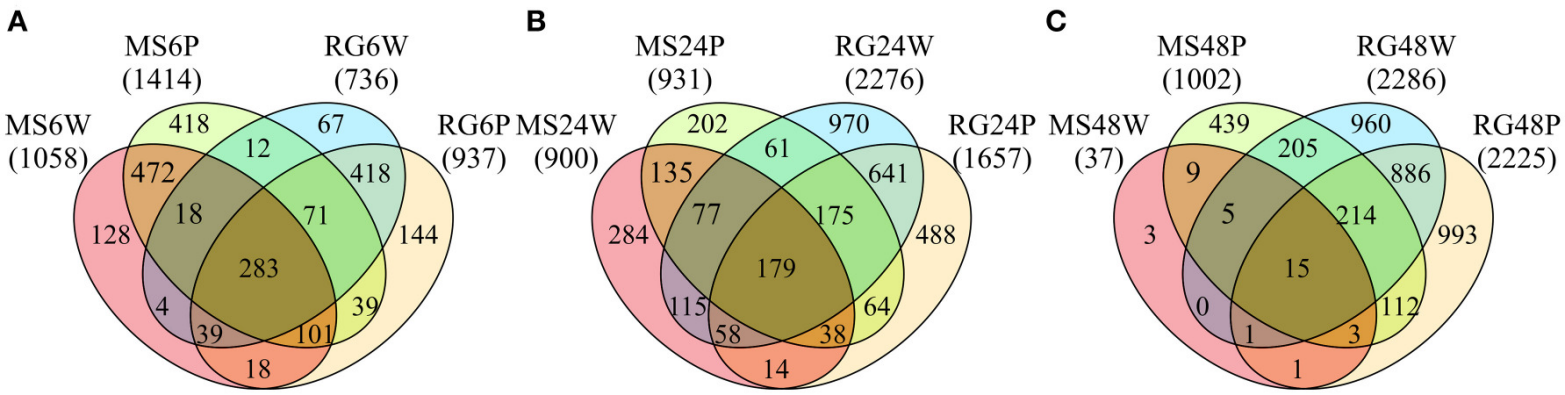
Venn diagrams showing the numbers of common and specific DE genes (FDR *p* value≤0.01) at 6 **(A)**, 24 **(B)**, and 48 **(C)** h after wounding (W) or *P. expansum* inoculation (P) in the parental lines *Malus sieversii–*PI613981 (MS) and *Malus* x *domestica* “Royal Gala” (RG).

Transcript analysis of MS and RG at T0 resulted in the detection of a total of 63,538 transcripts, 53,024 and 49,899 transcripts of which were assigned to MS Time 0 (MS0) and RG Time 0 (RG0), respectively. Despite the differences in the mapping of both parental lines against the reference genome (85.5 and 92.1% of the MS and RG clean reads, respectively), more genes were detected in the resistant genotype compared to the susceptible one. A total of 46,054 transcripts were common to both genotypes and a total of 2,561 genes were differentially expressed (DEGs: FDR adjusted *p* ≤ 0.01; Supplementary Table 4). MapMan analysis of the DEGs (adjusted *p* ≤ 0.01 and log_2_ ≥ 1 or ≤-1) was used to visualize the constitutive differences between both genotypes at time 0. DEGs assigned to “metabolic profile” related bins were involved in “minor CHO metabolism,” “TCA/org transformation,” “amino acid metabolism synthesis,” and “degradation,” among others. DEGs up-regulated in MS, however, were involved in “light reactions,” “ascorbate,” “gluthatione,” “tetrapyrrole,” “waxes,” and “sulfur-containing,” among others (data not shown). In general, all “biotic stress” pathways are represented in both parental lines (Supplementary Figure 6). Genes involved in “MYB-domain transcription factor family,” “jasmonate,” “ABA,” “secondary metabolites,” and “ethylene” showed higher expression in MS, however, “PR-proteins” and “brassinosteroid metabolism” were up-regulated in the RG. As shown in Table 2, all the genes coding for Myb-related proteins and 12 out of 13 genes coding for jasmonate-related proteins showed higher expression in the resistant genotype compared to the susceptible one. A similar pattern was observed for “ABA,” “secondary metabolites” and “ethylene” –related genes, where 75, 73, and 64% of the mapped genes showed stronger expression in the resistant genotype (Supplementary Table 5). In general, the expression at each time point after pathogen inoculation was higher in MS than in RG for almost all of these genes. However, 9 out of 17 genes coding for a putative disease resistance protein showed higher expression in the susceptible genotype at time 0, and this higher expression was maintained during the time course of infection (Table 2). In general, the DEGs identified at Time 0 highlight the fact that the MS and RG parents have very different genetic backgrounds, but some of these differences may account for the higher resistance of MS because genes classically associated with host resistance showed a higher expression in this genotype. Changes in transcript abundance with respect to T0 were analyzed within both parental lines in response to wounding and to *P. expansum* infection. Therefore, the data represent wound-specific and infection-specific responses within each genotype and not the constitutive differences between the genotypes. For both parental lines and at almost all time points, more genes were up-regulated than down-regulated in response to wounding (W) or wounding+infection (P) (Figure 2). When comparing time points and parental lines, the number of DEGs [FDR *p* ≤ 0.01, and log_2_(ratio MS/MS0 or ratio RG/RG0) ≥ 1 for up-regulation, and log_2_(ratio) ≤-1 for down-regulation] was higher in response to W and to P at 6 hpi in the MS genotype and at 24 and 48 hpi in the RG genotype. A total of 956 genes were differentially expressed in MS at 6 hpi, compared to 612 genes in RG at the same time point. The overlap in DEGs (FDR *p* ≤ 0.01) between treatments, time points, and genotypes was analyzed and is displayed in Venn diagrams (Figure 3). The expression of 283, 179, and 15 genes were common to both genotypes in response to wounding (W) or wounding–inoculation (P) at 6, 24, and 48 hpi, respectively. Based on the cluster analysis derived from the PCoA analysis and in the Venn diagrams, the highest number of DEGs was detected at 6 hpi in MS and at 48 hpi in RG. In the MS apples, 418 DEGs were uniquely expressed at 6 hpi in response to inoculation with *P. expansum*, while 128 DEGs were unique to wounding and 874 DEGs were shared in response to wounding and wounding/inoculation (Figure 3A). In contrast, 144 DEGs were unique to the RG genotype in response to inoculation, 67 in response to wounding and 811 DEGs were shared in response to wounding and wounding/inoculation. These data suggest a stronger and quicker response to wounding and wounding/inoculation in the MS genotype than in the RG genotype. At 24 and 48 hpi, the number of DEGs was much higher in the RG parent than in the MS parent. A total of 202 DEGs were specific to the response of MS to inoculation at 24 hpi, 284 DEGs were unique to the wounding response and 429 DEGs were shared in response to wounding and wounding/inoculation. In sharp contrast, 488 DEGs were unique to inoculation in the RG genotype, while 970 DEGs were unique to the wounding response in RG. A total of 1,053 DEGs in the RG genotype were shared at 24 hpi in response to wounding and wounding inoculation (Figure 3B). Only 3 DEGs were evident in the MS genotype at 48 hpi in response to wounding and 439 were specific to wounding/inoculation, while only 32 DEGs were shared between the two responses. In contrast, 960 DEGs were evident in the RG genotype at 48 hpi in response to wounding, 993 DEGs were evident in response to wounding/inoculation, and 926 DEGs were shared between the two responses (Figure 3C). Hierarchical cluster analysis and heat maps were created using the DEGs that are unique to wounding (Supplementary Figure 7) and inoculation with *P. expansum* (Supplementary Figure 8). The analysis of genes that are expressed specifically in MS along the time course of infection revealed that MS infected samples were clustered in two groups, one containing MS apples at 6 hpi and other group, divided in two subgroups, including MS apples infected 24 and 48 hpi (Supplementary Figure 8A). Two major groups are distinguished in RG infected apples, one containing two subgroups including samples at 6 and 24 hpi, and other group, at 48 hpi (Supplementary Figure 8B).

**Table 2:** Differentially expressed genes [FDR, *p* ≤ 0.01 and log_2_(RG0/MS0) ≥1 or ≤-1] coding for Myb–related proteins, pathogenesis–related proteins, resistance–related proteins and jasmonate (based on MapMan codes) in the resistant *M. sieversii* PI613981 (MS) and the susceptible “Royal Gala” (RG) parental lines at time 0.

**Feature ID**	**Description**	**MS0**	**MS6P**	**MS24P**	**MS48P**	**RG0**	**RG6P**	**RG24P**	**RG48P**
**MYB**									
MDP0000650225	Transcription factor WER	6.3	2.6	1.9	2.8	0.4	0.5	1.1	0.7
MDP0000275800	Transcription factor MYB44	18.7	14.1	7.1	5.5	2.7	5.4	2.7	4.7
MDP0000149535	Myb–related protein 306	6.8	1.3	1.5	2.0	1.1	0.3	1.4	0.9
MDP0000165715	Transcription factor MYB44	23.1	10.5	10.4	12.7	4.0	5.2	1.6	2.2
MDP0000187872	Transcription factor MYB44	17.1	6.4	7.8	12.6	3.5	14.0	5.8	4.8
MDP0000144744	Myb–related protein 306	37.7	16.9	24.6	23.2	12.4	9.1	1.3	0.9
MDP0000463846	Transcription factor MYB44	100.2	87.8	78.2	69.1	63.6	71.3	29.5	14.6
**PR–PROTEINS + DISEASE RESISTANCE**									
MDP0000213440	Probable protein Pop3	4.5	2.3	3.0	1.9	0.0	0.0	0.5	0.4
MDP0000474349	Pathogen–related protein	5.6	3.1	2.0	2.3	0.5	0.1	0.8	0.7
MDP0000788170	Pathogen–related protein	54.6	157.3	308.1	189.5	4.9	8.6	124.4	304.8
MDP0000686021	TMV resistance protein N	5.9	6.3	2.6	1.6	0.6	11.4	3.5	1.4
MDP0000688401	Pathogen–related protein	47.5	48.1	38.3	54.1	6.3	3.8	3.5	3.7
MDP0000799306	Pathogen–related protein	40.3	23.7	36.7	19.0	5.7	5.1	8.6	3.7
MDP0000253215	EDS1 (enhanced disease susceptibility 1)	13.6	18.0	14.5	14.1	3.3	5.1	8.0	5.6
MDP0000162236	EDS1 (enhanced disease susceptibility 1)	27.5	87.8	25.9	21.5	13.3	38.0	33.8	20.5
MDP0000300756	Disease resistance protein RPM1	7.1	4.9	4.3	2.5	26.1	21.3	4.4	2.2
MDP0000635659	Miraculin	11.3	5.7	5.8	4.9	75.4	50.9	11.2	7.3
MDP0000245324	Putative disease resistance protein At4g11170	4.4	4.6	3.7	3.3	42.9	50.9	27.1	14.1
MDP0000255700	Disease resistance protein RGA2	1.1	6.1	2.0	2.0	12.5	8.7	2.6	0.9
MDP0000125256	Disease resistance response protein 206	3.0	255.9	116.3	45.8	36.1	95.8	173.1	96.0
MDP0000119071	Graves disease carrier protein	2.7	0.7	2.3	1.6	33.5	3.0	2.5	1.1
MDP0000166759	Putative disease resistance protein RGA3	0.1	0.1	0.1	0.1	5.7	3.7	2.8	0.9
MDP0000827008	TMV resistance protein N	0.2	1.3	2.3	1.6	28.1	69.6	49.7	43.5
MDP0000640906	Probable disease resistance protein At5g66900	0.0	2.9	0.2	0.5	4.2	3.0	2.3	8.4
**JASMONATE**									
MDP0000300321	Lipoxygenase 2	15.4	3.1	6.1	5.4	0.2	0.1	1.1	1.2
MDP0000178268	Lipoxygenase 2.3	295.2	167.7	229.6	121.5	7.5	8.7	10.3	8.4
MDP0000225501	Allene oxide synthase	12.0	251.3	30.7	16.6	0.3	4.1	4.0	9.5
MDP0000174168	Lipoxygenase 2	16.1	10.1	16.1	10.1	0.9	1.4	4.0	3.1
MDP0000753547	Lipoxygenase 2	21.2	13.1	14.9	5.9	2.0	1.7	2.4	1.1
MDP0000274714	12–oxophytodienoate reductase 2	6.1	20.8	18.0	23.7	0.7	0.8	4.7	24.6
MDP0000169311	Lipoxygenase 2.3	45.1	33.9	84.6	46.1	5.5	7.2	28.1	17.7
MDP0000910857	Allene oxide synthase	7.0	271.6	34.3	17.8	1.0	97.1	137.8	234.1
MDP0000198152	Allene oxide synthase	12.3	3.3	8.2	4.8	3.7	1.3	1.4	0.8
MDP0000281525	Lipoxygenase 2	20.2	16.7	10.4	6.9	6.3	4.1	2.4	1.2
MDP0000150140	Allene oxide synthase	26.2	20.1	12.7	10.7	8.9	7.1	8.0	2.7
MDP0000424398	Allene oxide synthase	70.2	254.7	107.5	83.9	27.9	232.9	306.2	436.1
MDP0000450991	Lipoxygenase A	94.3	86.5	188.5	247.6	507.9	466.5	206.6	99.6

*Values represent the gene expression (in RPKM) at time 0 and at 6, 24 and 48 hours after P. expansum inoculation (P). Genes are ordered within each category from lower to higher log_2_(RG0/MS0)*.

**Figure 4:**
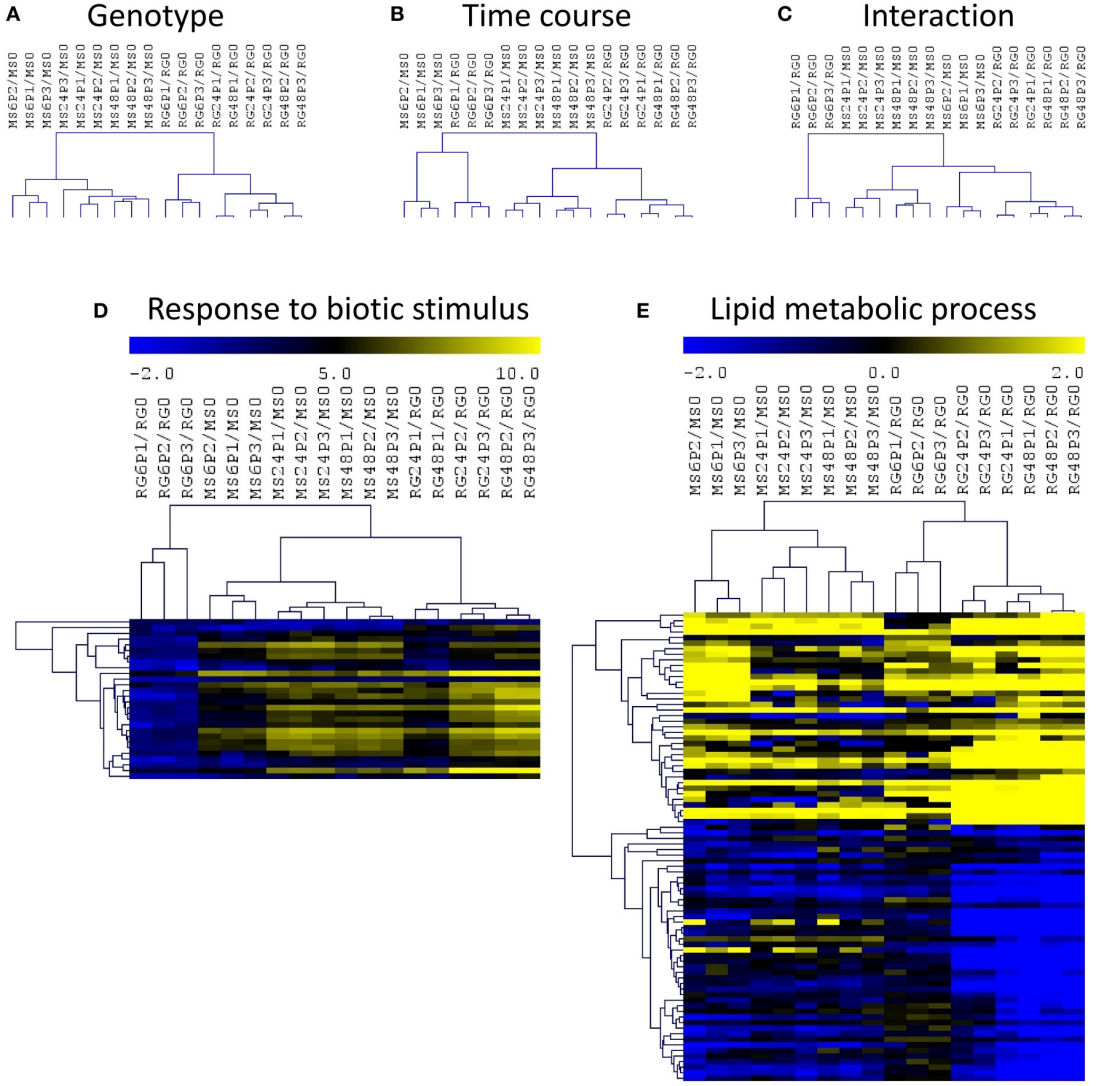
Hierarchical cluster (HCL) analysis and heatmaps of differentially expressed genes (two–factor ANOVA) based on genotype **(A)**, time course of *P. expansum* infection **(B)**, and the interaction **(C)**. Heatmap of genes included in the significant biological processes “response to biotic stimulus” **(D)** and “lipid metabolic process **(E)** based on the list of DEGs during the time course **(D)** or the interaction **(E)**. Abbreviations: *Malus sieversii*–PI613981 resistant apple (MS) and *Malus* x *domestica* “Royal Gala” susceptible apple (RG) at time 0 and inoculated with *Penicillium expansum* (P) at 6, 24, and 48 hpi. Data are expressed as log_2_(ratios).

In order to identify the interactions between genotypes and time course of infection, we have normalized each value by the expression level at time 0 for the corresponding genotype and we have analyzed those log_2_ (ratios) data using a two-factor ANOVA (Figure 4). This analysis and the corresponding hierarchical clustering (HCL) of the significant genes showed a clustering of the samples based on genotype (Figure 4A) and on the time course of the *P. expansum* infection (Figure 4B). The HCL of the DEGs in the interaction revealed the presence of a major cluster containing all the samples except RG6P/RG0. This major cluster was divided in two subclusters, one grouping the MS samples at 24 and 48 hpi and a second one grouping RG at 24 and 48 hpi and MS6P (Figure 4C). As shown in Figure 4C, the responses of the resistant MS genotype at 6 hpi were closer to those of the susceptible RG genotype at 24 and 48 hpi than the responses triggered in the MS at 24 or 48 hpi. Two–factor ANOVA revealed that the expression of 9,180, 4,347, and 3,297 genes was significant in each factor: genotype, time course and the interaction, respectively. No significant GO terms in the list of DEGs based on genotype were detected using SEA. However, four biological processes were significant in the time course condition: “response to biotic stimulus,” “single-organism metabolic process,” “defense response,” and “oxidation-reduction process.” The heat map of the “response to biotic stimulus” using the 28 DEGs included in this category revealed a lower expression level of all these genes in RG6P/RG0, whereas they all clustered independently in the other comparisons, were the expression levels were higher (Figure 4D). Out of 3297 DEGs based on the interaction, only the “lipid metabolic process” was significant (Figure 4E), and two major clusters of genes based on a high or a low expression level in all the samples analyzed were observed.

The same RPKM ratios normalized by time 0 were used to identify DEGs between both genotypes at each time point during the time course of infection [i.e., log_2_(MS6P/MS0) vs. log_2_(RG6P/RG0)]. The *t*-test analysis revealed that 4018 and 1082 genes were differentially expressed (FDR *p* ≤ 0.01) at 6 and 24 hpi. However, no significant GO terms were identified within both lists. At 48 hpi there were 2052 DEGs and 8 biological processes related to “reproduction,” “pollination,” and “cell recognition” were significant. An ANOVA test was done for each genotype along the time course of infection and the 349 DEGs [ANOVA of log_2_(MS6P/MS0 vs. log_2_(MS24P/MS0) vs. log_2_(MS48P/MS0); FDR *p* ≤ 0.01; Supplementary Table 6] in infected MS fruits were compared with the 417 DEGs [ANOVA of log_2_(RG6P/RG0 vs. log_2_(RG24P/RG0) vs. log_2_(RG48P/RGS0); FDR *p* ≤ 0.01; Supplementary Table 7] in the infected RG fruits. The comparison of the DEGs between both parental lines along the time course of infection showed that only 8 genes were common between both genotypes: MDP0000139683 (diacylglycerol kinase), MDP0000189486 (protein phosphatase 2C), MDP0000319964 (*Arabidopsis thaliana* seed gene 1, calcium ion binding), MDP0000622590 (HSF domain class transcription factor), MDP0000945267 (DNA binding), and three unknown proteins (MDP0000195437 and MDP0000563245). We have used SEA to obtain a list of enriched GO terms in both comparisons. Two categories, “response to biotic stimulus” and “defense response,” were enriched in the resistant MS genotype (Supplementary Figure 9). The genes associated with these categories were three genes coding for major allergen Mal d 1 protein (MDP0000295540, MDP0000312569, MDP0000533638), one coding for a pathogenesis-related protein PR (MDP0000782085), one coding for a ribonuclease-like PR protein (MDP0000831518), and one conserved unknown protein (MDP0000889787). However, no significant terms were obtained for the infected susceptible RG fruits.

Since the QTL analysis of the GMAL4593 mapping population (Norelli et al., 2017) indicated the presence of a significant QTL for blue mold resistance on LG3, that was contributed by the MS parent, special attention was given to the DEGs that mapped to the qM–*Pe*3.1 QTL (*M* × *d* v.1: 26,848,396–28,424,055) on LG3. To determine the DEGs (FDR *p* ≤ 0.01) on LG3, we performed a Baggerley's test in the comparison log_2_(RG0/MS0) and an ANOVA test among log_2_(MS6P/MS0), log_2_(MS24P/MS0), log_2_(MS48P/MS0), log_2_(RG6P/RG0), log_2_(RG24P/RG0), and log_2_(RG48P/RG0) comparisons (Table 3). Out of 20 DEGs that mapped to the qM-*Pe*3.1 QTL on LG3, 17 genes were more highly expressed in the resistant parent MS genotype than in the RG susceptible genotype at time 0. Five of them were selected to confirm their higher expression in MS0 using RT-qPCR (Supplementary Figure 10). Pearson correlation coefficients between reads per kilobase per million mapped reads (RPKM) values from RNA-Seq and relative gene expression (RGE) values from RT-qPCR of the assayed genes ranged between 0.70 (for MDP0000494903) and 0.89 (for MDP000133552), with an average value of 0.78.

**Table 3:** Differentially expressed genes (FDR, *p* ≤ 0.01) in the resistant *M. sieversii* PI613981 (MS) and the susceptible “Royal Gala” (RG) parental lines at time 0 and at 6, 24, and 48 hpi (samples wounded and infected with *P. expansum*, denoted as “P”) that mapped to the qM–*Pe3*.1 QTL on LG3.

	**Description**	**log_2_(RG0/MS0)**	**log_2_(MS6P/MS0)**	**log_2_ (MS24P/MS0)**	**log_2_ (MS48P/MS0)**	**log_2_ (RG6P/RG0)**	**log_2_ (RG24P/RG0)**	**log_2_ (RG48P/RG0)**
MDP0000133552		−3.2	1.6	1.4	0.9	0.6	3.2	2.0
MDP0000134935	Homogentisate 1,2-dioxygenase	−0.5	−1.3	−0.7	−0.5	−0.7	−1.5	−2.5
MDP0000182835		+	2.1	–	−0.8	NaN	+	+
MDP0000194902	E3 ubiquitin-protein ligase RMA1H1	−1.8	−1.4	−0.8	−0.4	−0.9	−0.8	0.0
MDP0000194903	Thioredoxin M4, chloroplastic	−2.3	−1.9	−0.6	−0.3	−0.4	0.2	−2.6
MDP0000237909	Cytochrome c oxidase copper chaperone	0.9	0.1	−0.1	−2.5	−0.6	−2.4	−3.8
MDP0000243585	Metallothionein-like protein type 3	−5.8	−1.1	−1.4	−0.1	−0.7	−0.4	−0.2
MDP0000271115	Lectin–domain containing receptor kinase A4.2	+	–	−1.7	−0.6	+	+	+
MDP0000285282	Monoglyceride lipase	−1.3	−1.2	−0.5	0.0	−0.1	0.6	0.7
MDP0000301516	Probable ubiquitin–conjugating enzyme E2 26	0.7	−0.6	−0.5	−0.4	−0.6	−1.3	−2.0
MDP0000324718	Ethylene-responsive transcription factor 4	−2.7	−0.7	−2.2	−1.7	−0.8	−2.0	−1.1
MDP0000357502		+	−0.1	−0.9	−0.6	+	+	+
MDP0000367430		+	4.5	4.6	3.4	+	+	+
MDP0000368270		+	–	–	–	NaN	+	+
MDP0000407067	Secologanin synthase	+	6.0	5.3	5.6	+	+	+
MDP0000473430	Threonine synthase 1, chloroplastic	+	–	0.5	–	NaN	+	+
MDP0000573244	Probable protein phosphatase 2C 39	0.7	0.3	−0.2	−0.3	−0.4	−2.3	−2.4
MDP0000902023	Lectin–domain containing receptor kinase A4.3	+	–	–	–	NaN	+	+
MDP0000915514		−2.4	3.1	3.9	1.7	–	3.6	4.0
MDP0000921179	Uncharacterized basic helix–loop–helix protein At1g06150	−4.4	−1.7	0.7	0.9	1.4	5.6	4.2

*The symbol “+” indicates no expression in the denominator, “–“ indicates no expression in the numerator, “NaN” indicates no expression detected in any of the samples*.

**Figure 5:**
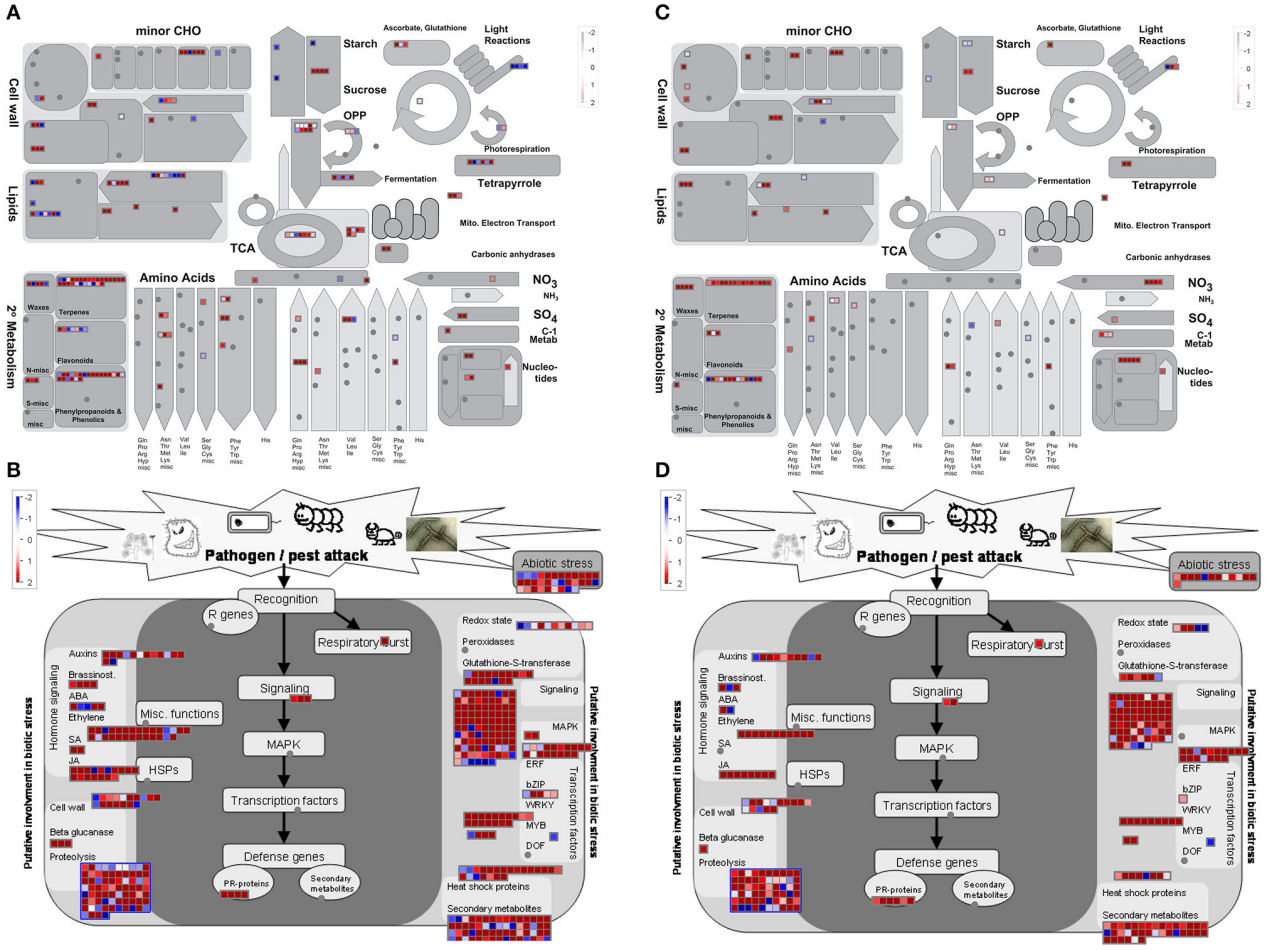
Overview of metabolic pathways **(A,C)** and of genes related to biotic stress **(B,D)** for each DEGs upon *P. expansum* infection after 6 hpi in the resistant MS parent **(A,B)** or in the susceptible RG parent **(B,D)** vs. Time 0. The scale bar displays changes in gene expression as log_2_ (ratio MS6P/MS0) or log_2_(ratio RG6P/RG0) that were significant (FDR *p* value≤0.01). Genes induced due to the *P. expansum* infection are highlighted in red and repressed genes are highlighted in blue. CHO, carbohydrates; OPP, oxidative pentose phosphate; TCA: tricarboxylic acid cycle.

MapMan software was used to provide an overview of genes modulated in the main metabolic pathways in the two parental lines in response to *P. expansum* inoculation. DEGs at 6, 24, and 48 hpi vs. time 0 were binned to MapMan functional categories in both parental lines (Figure 5, and Supplementary Figures 11, 12, respectively). The expression of genes involved in “lipid metabolism,” “cell wall,” “light reactions,” and “waxes” decreased at 6, 24, and 48 hpi in MS compared to Time 0, whereas an increase in “TCA/org transformation,” and “amino acid degradation” was observed. However, the expression of genes involved in almost all the metabolic pathways was decreased during the time course of the infection compared to healthy tissue in the susceptible RG genotype. Similar results were observed for the biotic stress pathways, where the expression of more genes was increased in the resistant parent at 6 hpi compared to healthy tissue than in the susceptible parent. It is important to note the decrease in the expression level of a high amount of genes related to biotic stress in the susceptible genotype at 48 hpi compared to Time 0 and to the resistant genotype (Supplementary Figure 12).

**Figure 6:**
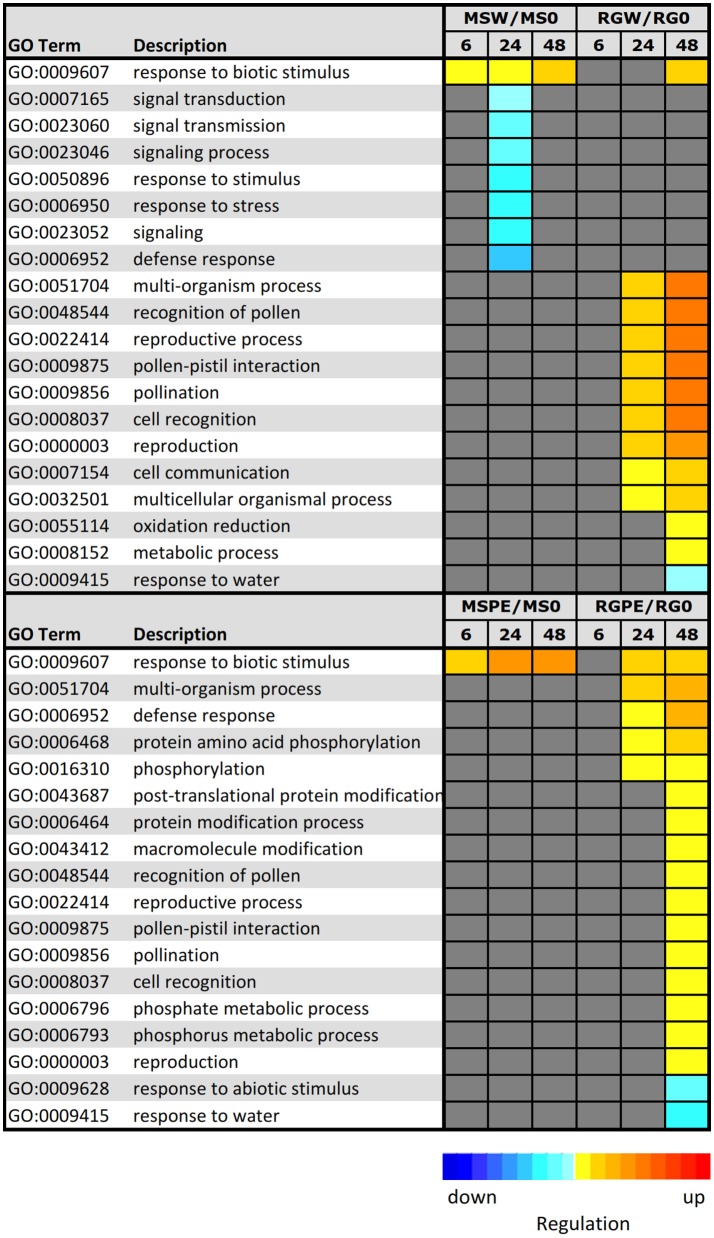
Parametric analysis of gene–set enrichment (PAGE) using AgriGo to identify enriched GO terms onto the biological term inclusive of gene expression levels. The colored blocks represent the level of up/downregulation of each term at a certain time–point. The yellow–to–red, cyan–to–blue, and grayscale represent the term is upregulated, downregulated, or no–significant change, respectively. The adjusted *p*-value of the term determines the degree of color saturation of the corresponding box. MSW/MS0 = comparison of *M. sieversii* at 6, 12, and 48 h after wounding, relative to Time 0. RGW/RG0 = comparison of “Royal Gala” at 6, 12, and 48 h after wounding, relative to Time 0. MSPE/MS0 = comparison of *M. sieversii* at 6, 12, and 48 h post-inoculation with *P. expansum*, relative to Time 0. RGPE/RG0 = comparison of “Royal Gala” at 6, 12, and 48 h post–inoculation with *P. expansum*, relative to Time 0.

In order to elucidate the key processes that were altered in infected samples, an analysis of functional enrichment categories in the set of DEGs was conducted (Figure 6). Parametric Analysis of Gene Set Enrichment (PAGE) tool included in the AgriGO website was used to facilitate the global analysis of gene expression, with the significantly differentially expressed genes assigned to different functional categories. A total of 7 GO terms, including “defense response,” “response to stress,” and “signal transduction,” were down-regulated at 24 h after the resistant MS parent was wounded. PAGE analysis also showed that DEGs in infected MS during the time course were mainly involved in only one biological process associated with “response to biotic stimulus (GO:0009607),” that was also up-regulated in the resistant parent in response to wounding. Genes included in this term are pathogenesis-related (PR) protein encoding genes, such as the major allergen Mal d 1 and MLP-like proteins. Different GO terms were up-regulated in RG after wounding or after infection: “multi-organism process,” “reproductive process” and processed related to pollination, among others. Moreover, processes related to defense response and to protein modifications (“protein amino acid phosphorylation,” “protein modification process” and others) were up-regulated at 48 hpi in the infected, susceptible RG genotype. Only two biological processes were down-regulated at 48 hpi in the inoculated RG genotype: “response to abiotic stimulus” and “response to water.”

## Discussion

In the present work, the parents of the mapping population used by Norelli et al. (2017) to identify the qM-*Pe*3.1 QTL for blue mold resistance were used to elucidate the molecular basis underlying the resistance to *P. expansum* (a necrotrophic pathogen) infection observed in *M. sieversii*. Our working hypothesis is that the higher resistance of the MS genotype must be due either to a higher constitutive basal defense system and/or to a faster and/or stronger induction of an effective defense response triggered upon *P. expansum* inoculation. To test the first possibility, a direct comparison between both genotypes needs to be conducted. Although, read mapping efficiency against the *Malus* × *domestica* reference genome was slightly lower in MS than in RG (85.5 vs. 92.1%), this difference does not preclude a direct comparison between both datasets. While comparing the transcriptome of a domesticated apple variety, RG, with a wild progenitor species, *M. sieversii*–PI613981, can be problematic, this approach has been used to compare gene expression in different tissues or stresses of wild and cultivated tomatoes, potatoes and watermelons (Chen et al., 2015; Guo et al., 2015; Zuluaga et al., 2015; Dai et al., 2017). In each case, valuable information has been gained into the processes being examined. We are aware that these two genotypes are highly heterozygous and besides their different susceptibility to blue mold infection, they also differ in their resistance to several diseases, including apple scab, fire blight, and in many other phenotypic traits. Thus, differences in gene expression levels between MS and RG at time 0 may reflect all these differences. However, if resistance to blue mold infection is already activated in MS before pathogen inoculation, it could also be reflected at the gene expression level. To test the second hypothesis, we compared wound- and pathogen-induced responses in each genotype by comparing untreated samples (Time 0) of MS and RG to their respective samples 6, 24, and 48 h following wounding (W) or inoculation with *P. expansum* (P). These DEGs were informative of specific wound and host responses within resistant MS or susceptible RG. Of particular importance was the identification of DEGs in the susceptible RG and resistant MS parents during the early (up to 48 h after inoculation) stages of infection and decay development.

The results of the current study demonstrate that significant differences in gene expression exist in the resistant *M. sieversii*–PI613981 and the susceptible cultivar RG in untreated tissues at Time 0, and in response to just wounding, as well as wounding and inoculation with *P. expansum*. In some plant-pathogen systems, the accumulation of relatively high levels of reactive oxygen species (ROS) produced by either the plant or/and the pathogen results in cell death. In the case of necrotrophic fungi, this response can be utilized to establish induced susceptibility rather than induced resistance since the presence of nutrients resulting from the dead cells allows the pathogen to establish itself and develop further decay (Heller and Tudzynski, 2011). In order to prevent the accumulation of ROS, an increase in gene expression of genes encoding ROS-detoxifying enzymes, such superoxide dismutase, ascorbate peroxidase, and peroxidase, has been observed in apples inoculated with *P. expansum* (Vilanova et al., 2014). Related to this, the current work indicated that the glutathione-ascorbate cycle, that detoxifies hydrogen peroxide and the secondary metabolism by–products related to waxes, were more highly expressed in the resistant MS parental line, even at time 0. As mentioned before, interpretation of the function of DEGs at Time 0 in relation to blue mold resistance, however, is problematic. In addition to qM-*Pe*3.1, MS carries genetic determinants associated with resistance to *Venturia inaequalis* and *Erwinia amylovora* (unpublished data). Therefore, no specific conclusions can be drawn from DGEs at time 0, when no pathogens are present. What was readily apparent in the results of this study is that the resistant genotype underwent a more rapid response than RG, the susceptible genotype, to both wounding and wounding plus inoculation with *P. expansum*. This premise is supported by the Venn diagrams (Figure 3) indicating the number of unique and shared DEGs.

In the current study, ethylene-related genes were more highly upregulated in the resistant MS genotype. In a related study, Logemann et al. (2013) reported that *PROPEP* genes in *Arabidopis* code for small proteins that act as DAMPS in the response of *Arabidopsis* to *Botrytis cinerea*. Liu et al. (2013) demonstrated that a pepr1/pepr2 mutant exhibited reduced levels of ethylene as well as susceptibility to *B. cinerea*. In our study, we also found that genes involved in “jasmonate,” and “MYB-domain transcription factor family” were up-regulated in MS. Jasmonate and MYB proteins may play a role in enhancing fruit tissue resistance responses to wounding and pathogen attack as JA and ethylene dependent defense responses are known to be induced when cell wall integrity is modified (Ellis and Turner, 2001; Ellis et al., 2002; Zhong et al., 2002).

The MYB family of proteins is large, functionally diverse, and represented in all eukaryotes. Most MYB proteins function as transcription factors with varying numbers of MYB domain repeats conferring their ability to bind DNA. They are widely distributed in plants and have been implicated in ABA response and also interact with other transcription factors (Ambawat et al., 2013). MYB genes have been extensively studied and members of the MYB family have been found to be involved in a variety of biological functions like phenylpropanoid metabolism (Grotewold, 2006; Hichri et al., 2011), and biotic and abiotic stress response (Lippold et al., 2009; Segarra et al., 2009). The family of R2R3-MYB-like transcription factors has repeatedly been implicated in JA dependent defense responses. For instance, the *OsLTR1* gene from rice regulated JA-dependent defense whereas AtMYB15, AtMYB34, AtMYB51, and AtMYB75 were associated with the wound response or resistance against insect herbivores (Cheong et al., 2002; Johnson and Dowd, 2004).

RNA-Seq was used in the present study to characterize the expression of genes associated with response to wounding or to wounding and *P. expansum* infection, after 6, 24, and 48 hpi. The low number of DEGs found to co-locate within the genomic region mapped to qM-*Pe*3.1 suggests that the effect of the QTL is not due to an accumulation of genes involved in host responses to *P. expansum*, however, there is the possibility that the DEGs may be controlled in trans by regulatory elements present in the regions of interest. SEA and PAGE analyses identified enriched GO terms associated with the biological term “response to biotic stimulus” in the resistant parent in response to wounding or wounding-infection (Figure 6). This term is enriched in several pathogenesis-related proteins, such as the major allergens Mal d 1, Pru ar 1, and Pru av 1. These proteins belong to the PR-10 class of proteins, and they are induced under various stress conditions and act as common allergens. The biological function of PR-10 proteins is not entirely known, but it has been suggested that these proteins possess ribonuclease activity, which may prevent fungal growth in host plants (Sinha et al., 2014). In response to the pathogen, the major allergens represented by the term “response to biotic stimulus” were induced in the resistant parent to a higher level compared to the wounding response, and with a faster response at 6 hpi. The fact that these Mal d 1 proteins may be associated with an early resistance response has been described previously by Buron-Moles et al. (2015).

Within the group of genes located in the qM-*Pe*3.1 QTL on LG3 (Table 3), a metallothionein-like protein encoding gene (MDP0000243585) showed higher expression in MS compared to RG at time 0, and also during the time course of the infection. The constitutive expression of metallothionein-like protein encoding genes has been described previously in uninfected apple leaves from a resistant apple cultivar and in leaves inoculated with *Venturia inaequalis* (Degenhardt et al., 2005), and in plant cells after a pathogen attack (Xuxia et al., 2012). The role of metallothioneins in response to biotic stress is not fully understood. It has been suggested that these small proteins are involved in essential metal homeostasis and (toxic) metal detoxification, especially cadmium, copper, zinc and mercury (Cobbett and Goldsbrough, 2002). Thus, these proteins may inhibit fungal growth through metal ion sequestration. These proteins can also protect against oxidative damage and other abiotic stresses which result in generation of ROS (Tripathi et al., 2015). Other genes located on LG3 that exhibited significant up–regulation in MS after pathogen inoculation are MDP0000133552, coding for a late embryogenesis abundant (LEA) protein containing a harpin–inducible domain, and MDP0000324718, which codes for an ethylene–responsive transcription factor. Additional genes showing strong up–regulation in MS following pathogen inoculation are MDP0000357502, coding for an unknown protein, and MDP0000407067, annotated as a secologanin synthase, MDP000019402, coding for a E3 ubiquitin–protein ligase, MDE0000194903, coding for a thioredoxin, MDP0000243585, coding for a metallothionein–like protein, and MDP0000285282, coding for a monoglyceride lipase. These genes represent potential candidate genes responsible for the observed resistance in the MS genotype, however, it also possible that genes responsible for the observed resistance were not detected by the RNA-Seq analysis, or that DEGs may be controlled in trans by regulatory genes in the region. This may be attributed to several factors. The alleles of the contributing resistance gene(s) may contain only a few base pair differences between the two genotypes that would not be discerned by RNA-Seq analysis. The timing of the sampling and/or the sensitivity of the RNA-seq assay may not have been adequate to reveal the specific gene(s) responsible for the resistance QTL, or the resistance alleles could function in pathogen recognition, rather than induced resistance responses, which may not be detected as DEGs in RNA-Seq. In all cases, more detailed genetic analyses will need to be conducted.

In conclusion, the timing of DEGs, along with the phenotypic response to wounding/inoculation in the two genotypes, indicate that the resistant MS (PI613981) genotype shows both a higher basal level of resistance and a more rapid response to the presence of the pathogen than the RG genotype. This rapid response was followed by a wound response that compartmentalized the injury and prevented *P. expansum* from becoming established. The rapid activation of genes related to wound healing processes, including the deposition of a structural barrier, PR-proteins, and phytoalexins, may play a major role in blue mold resistance observed in MS (PI613981). Janisiewicz et al. (2016) examined wound response in several accessions of *M. sieversii* that were categorized as being immune/resistant, moderately resistant, and susceptible. Their results also suggested that resistance was associated with several mechanisms related to wound response. GO and KEGG analyses suggest that the activation of resistance-related genes may be modulated by the jasmonic acid and ethylene signaling system triggered by wounding. The large number of DEGs evident in the RG genotype at 24 and 48 h after inoculation may not be associated with an active “resistance” response but rather reflect changes in the cellular metabolism and biochemistry that occur as the infection of apple tissues by *P. expansum* becomes established, cells began to senesce and or die, and lesions develop. Host response to necrotrophic pathogens is complex and depends on the nature of the virulence mechanism exhibited by the pathogen. As reviewed by Wang et al. (2014) a large number of pathogen- and host-derived molecules may mediate the infection process and determine a resistant or susceptible response. While not definitive, the current study, along with the genetic study conducted by Norelli et al. (2017) provide an excellent basis for conducting more detailed studies on the resistance to blue mold exhibited by the *M. sieversii* genotype, PI613981.

## Author contributions

A-RB was involved in the data analysis and writing. JN was involved in the project design, data analysis and writing. EB was involved in the construction of the libraries, and RT PCR analyses. AA was involved in the PCoA analysis and interpretation of the results. EL was involved in the data analysis and interpretation. LG-C was involved in data interpretation and writing the manuscript. MW and SD were involved in the project design, supervision, data analysis, and writing.

### Conflict of interest statement

The authors declare that the research was conducted in the absence of any commercial or financial relationships that could be construed as a potential conflict of interest.
