# Supplementary material for: Transcriptomic Response of Resistant (PI613981–Malus sieversii) and Susceptible (“Royal Gala”) Genotypes of Apple to Blue Mold (Penicillium expansum) Infection
Source: Front Plant Sci. 2017 Nov 16;8:1981. doi: 10.3389/fpls.2017.01981 (PMC5696741; doi:10.3389/fpls.2017.01981)
Supplement: Supplementary file 5 [file Presentation1.PDF]

Supplementary Material

Transcriptomic response of resistant (PI613981 – *Malus sieversii*) and susceptible ('Royal Gala') parents of the GMAL4593 mapping population of apple to blue mold (*Penicillium expansum*) infection

Ana-Rosa Ballester[1*], John Norelli[2], Erik Burchard[2], Ahmed Abdelfattah[3], Elena Levin[4], Luis González-Candelas[1], Samir Droby[4], and Michael Wisniewski[2*]

[1]Instituto de Agroquímica y Tecnología de Alimentos (IATA-CSIC), Calle Agustín Escardino Benlloch 7, Paterna, Valencia 46980, Spain
[2]U.S. Department of Agriculture – Agricultural Research Service (USDA-ARS), 2217 Wiltshire Road, Kearneysville, WV 25430, USA
[3]Dipartimento di Agraria, Università Mediterranea di Reggio Calabria, Località Feo di Vito, Reggio Calabria, 89122, Italy
[4]Agricultural Research Organization (ARO), The Volcani Center, P.O. Box 6, Bet Dagan, Israel

*** Correspondence:** Michael Wisniewski, michael.wisniewski@ars.usda.gov; Ana-Rosa Ballester, ballesterar@iata.csic.es

Supplementary Table 1. Gene-specific primer sequences used to conduct RT-qPCR analyses of five selected differentially expressed genes in the different treatments and one reference gene.

Gene	Description	Forward (5'→3')	Reverse (5'→3')
MDP0000060858 (FYPP3)	phytochrome-associated protein phosphatase 3	TCGAGCACACCAACTTGTTC	GATAGAAGCCACATTCCCACA
MDP0000133552	conserved unknown protein	GCAAAGGGTAGCGTGAAA	CACTCCCTAAACCACCAA
MDP0000357502	hypothetical protein	CATCCAAAGCCCCCATCT	CTTTTCTCTCTGTGTTGGT
MDP0000194902	E3 ubiquitin-protein ligase RMA1H1	ACTGGTTCCTTTGACTGC	ACTGAAGAAGGGGAGATG
MDP0000194903	Thioredoxin M4, chloroplastic	AAGCAATATGTTGGGAAGC	GTGGTTAATGTGGATTTGGG
MDP0000407067	Secologanin synthase	ATGGTGAGATTGATCTGGT	TCGCCTCTTCCCTTTTGT

Supplementary Table 2. Weight, starch and firmness in the resistant *M. sieversii* (MS) and in the susceptible 'Royal Gala' (RG) parental lines. Relative starch content was determined using a starch-iodine index chart for 'Golden Delicious'. Values represents the mean ± standard deviation (n = 5).

Line	Total Weight (n=5)	Weight (n=1)	Relative Starch Content	Firmness
MS	117.60	23.52	8.60 ± 0.55	83.71 ± 7.02
RG	417.23	83.45	7.60 ± 0.89	90.92 ± 6.49

Supplementary Table 3. Mapping characteristics of *Malus sieversii* PI613981 (MS) and 'Royal Gala' (RG) parental lines for each sample analyzed (healthy tissue at time 0, wounded and wounded-inoculated tissues at 6, 24 and 48 hpi) to the reference genome *Malus* x *domestica* v1.0. Reads were deposited in the NCBI Sequence Read Archive (SRA) accession ID SRP105163 (BioProject ID PRJNA383305).

Sample	Biosample ID (NCBI)	No. Total Reads	No. (and %) clean reads		No. (and %) reads mapped onto the *M. domestica* v1.0		Number of identified genes
MS01	SAMN06765572	7,712,047	6,927,812	(89.8%)	5,884,191	(84.9%)	42,365
MS02	SAMN06765573	8,962,310	8,078,780	(90.1%)	7,398,939	(91.6%)	46,411
MS03	SAMN06765574	5,907,863	5,292,332	(89.6%)	4,705,185	(88.9%)	39,958
MS6W1	SAMN06765575	5,439,124	4,883,475	(89.8%)	4,536,362	(92.9%)	39,259
MS6W2	SAMN06765576	6,350,564	5,658,370	(89.1%)	4,866,139	(86.0%)	42,414
MS6W3	SAMN06765577	5,723,912	5,127,128	(89.6%)	3,844,105	(75.0%)	41,322
MS24W1	SAMN06765578	6,813,540	5,649,577	(82.9%)	4,175,921	(73.9%)	39,019
MS24W2	SAMN06765579	6,907,817	5,773,812	(83.6%)	5,008,413	(86.7%)	39,884
MS24W3	SAMN06765580	5,471,065	4,561,423	(83.4%)	3,766,381	(82.6%)	37,792
MS48W1	SAMN06765581	9,604,304	9,186,364	(95.6%)	8,903,325	(96.9%)	43,491
MS48W2	SAMN06765582	5,968,122	5,709,467	(95.7%)	4,653,095	(81.5%)	45,143
MS48W3	SAMN06765583	6,728,925	5,559,059	(82.6%)	4,609,661	(82.9%)	43,538
MS6P1	SAMN06765584	6,857,144	6,156,699	(89.8%)	5,622,418	(91.3%)	38,972
MS6P2	SAMN06765585	6,007,796	5,381,946	(89.6%)	4,553,232	(84.6%)	48,761
MS6P3	SAMN06765586	11,303,299	10,108,656	(89.4%)	8,999,132	(89.0%)	45,766
MS24P1	SAMN06765587	8,235,011	6,862,053	(83.3%)	6,053,501	(88.2%)	42,179
MS24P2	SAMN06765588	6,787,493	5,630,056	(82.9%)	4,825,471	(85.7%)	40,534
MS24P3	SAMN06765589	7,397,163	6,134,832	(82.9%)	5,083,227	(82.9%)	47,728
MS48P1	SAMN06765590	7,035,155	5,793,596	(82.4%)	4,686,240	(80.9%)	42,697
MS48P2	SAMN06765591	6,724,355	5,491,129	(81.7%)	4,343,155	(79.1%)	42,333
MS48P3	SAMN06765592	6,551,788	5,390,287	(82.3%)	4,089,047	(75.9%)	40,055
RG01	SAMN06765593	6,428,600	5,762,277	(89.6%)	5,535,102	(96.1%)	42,635
RG02	SAMN06765594	4,564,090	4,112,813	(90.1%)	3,964,977	(96.4%)	35,793
RG03	SAMN06765595	8,067,379	7,231,701	(89.6%)	6,960,192	(96.2%)	40,793
RG6W1	SAMN06765596	6,566,245	5,895,179	(89.8%)	5,631,412	(95.5%)	37,657
RG6W2	SAMN06765597	7,660,431	6,875,448	(89.8%)	6,598,844	(96.0%)	40,313
RG6W3	SAMN06765598	6,692,431	5,978,789	(89.3%)	5,601,528	(93.7%)	38,960
RG24W1	SAMN06765599	6,453,947	5,376,994	(83.3%)	5,012,248	(93.2%)	40,700
RG24W2	SAMN06765600	7,534,303	6,298,786	(83.6%)	5,863,014	(93.1%)	41,366
RG24W3	SAMN06765601	8,227,345	6,822,609	(82.9%)	6,573,236	(96.3%)	41,837
RG48W1	SAMN06765602	8,538,817	7,050,736	(82.6%)	6,241,215	(88.5%)	46,031

RG48W2	SAMN06765603	6,788,501	5,673,117	(83.6%)	5,104,352	(90.0%)	40,687
RG48W3	SAMN06765604	7,255,985	6,035,988	(83.2%)	4,881,205	(80.9%)	39,923
RG6P1	SAMN06765605	6,428,688	5,779,922	(89.9%)	5,482,229	(94.8%)	38,937
RG6P2	SAMN06765606	7,464,908	6,723,654	(90.1%)	6,530,424	(97.1%)	40,309
RG6P3	SAMN06765607	6,925,772	6,218,591	(89.8%)	5,971,948	(96.0%)	40,101
RG24P1	SAMN06765608	6,775,441	5,701,738	(84.2%)	4,820,158	(84.5%)	58,277
RG24P2	SAMN06765609	7,866,272	6,544,673	(83.2%)	5,721,413	(87.4%)	41,284
RG24P3	SAMN06765610	7,863,952	6,531,722	(83.1%)	5,992,734	(91.7%)	41,766
RG48P1	SAMN06765611	16,550,262	13,842,815	(83.6%)	12,311,014	(88.9%)	61,490
RG48P2	SAMN06765612	8,444,278	6,920,649	(82.0%)	6,238,772	(90.1%)	42,010
RG48P3	SAMN06765613	7,102,234	5,806,622	(81.8%)	5,245,293	(90.3%)	41,299

Supplementary Table 4. Differentially expressed genes between MS and RG at time 0.

(Excel file)

Supplementary Table 5. Differentially expressed genes (FDR, p value≤0.01 and $\log_2$(RG0/MS0) ≥1 or ≤-1) coding for ABA, secondary metabolites and ethylene -related proteins (based on MapMan codes) in the resistant *M. sieversii* PI613981 (MS) and the susceptible 'Royal Gala' (RG) parental lines at time 0. Values represent the gene expression (in RPKM) at time 0 and at 6, 24 and 48 hours after *P. expansum* inoculation (P). Genes are ordered within each category from lower to higher $\log_2$(RG0/MS0).

(Excel file)

Supplementary Table 6. DEGS for infected MS fruits were obtained by an analysis of variance (ANOVA) of $\log_2$(MS6P/MS0 vs $\log_2$(MS24P/MS0) vs $\log_2$(MS48P/MS0) with a FDR p value≤0.01. Values represent RPKM data.

(Excel file)

Supplementary Table 7. DEGS for infected RG fruits were obtained by an analysis of variance (ANOVA) of $\log_2$(RG6P/RG0 vs $\log_2$(RG24P/RG0) vs $\log_2$(RG48P/RGS0) with a FDR p value≤0.01. Values represent RPKM data.

(Excel file)

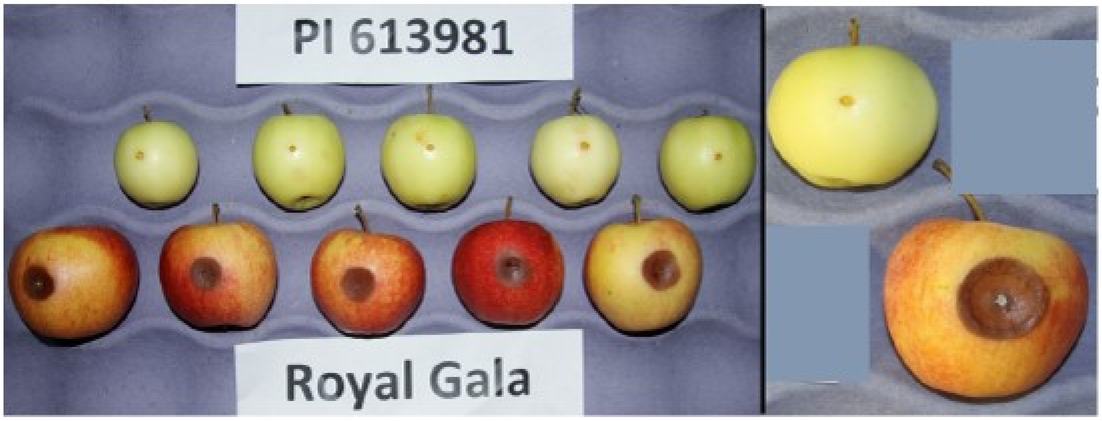

Supplementary Figure 1. Five *Malus sieversii*-PI613981 resistant apples and five *Malus* × *domestica* ‘Royal Gala’ susceptible apples inoculated with *Penicillium expansum*.

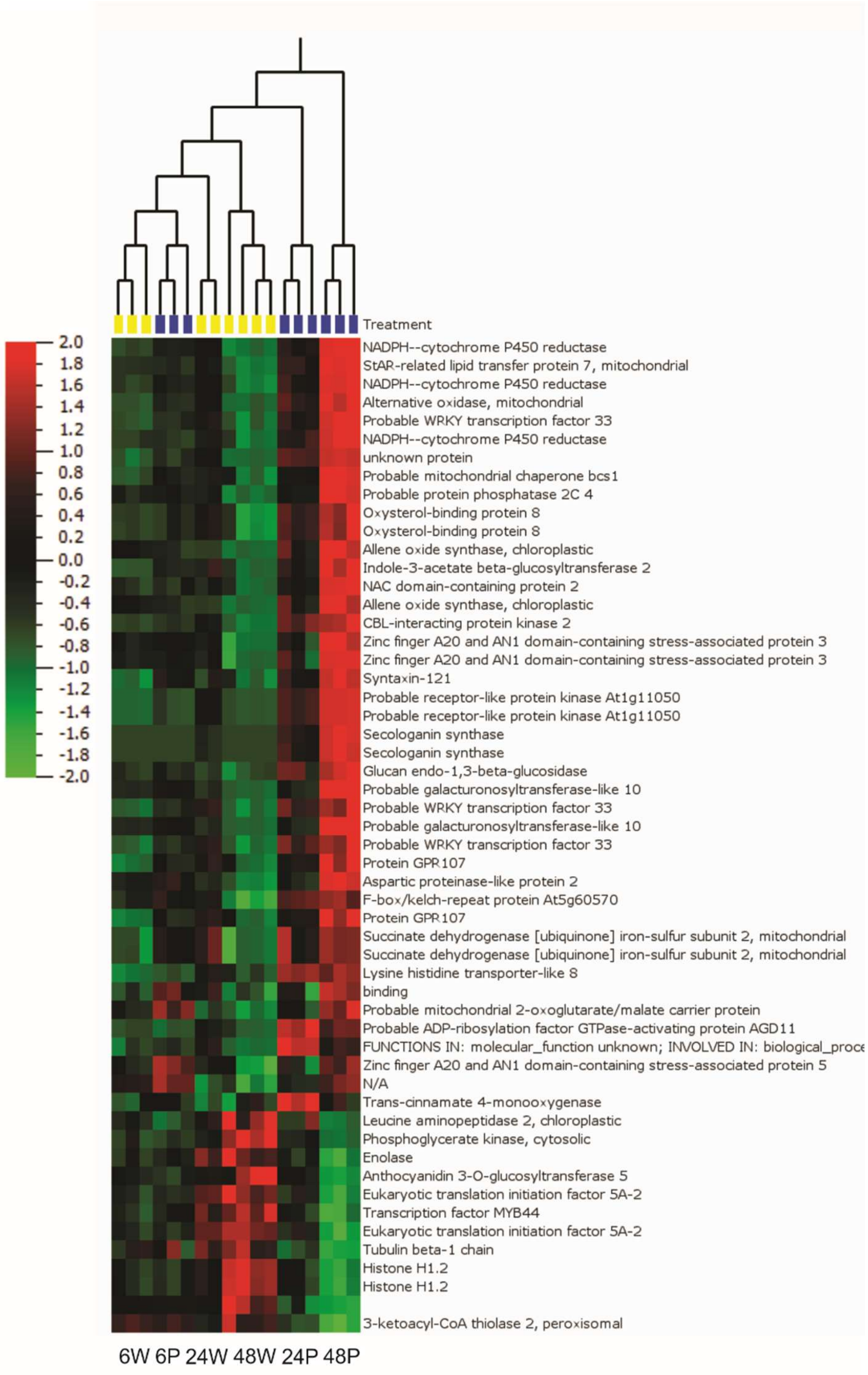

Supplementary Figure 2. Hierarchical clustering of differentially expressed genes in *Malus* × *domestica* 'Royal Gala' (RG) apples at 6, 24, and 48 hours after wounding (W; yellow marked lanes) or wounding/inoculation with *P. expansum* (P; blue marked lanes) Each lane represents an independent biological replicate ($p \leq 0.01$).

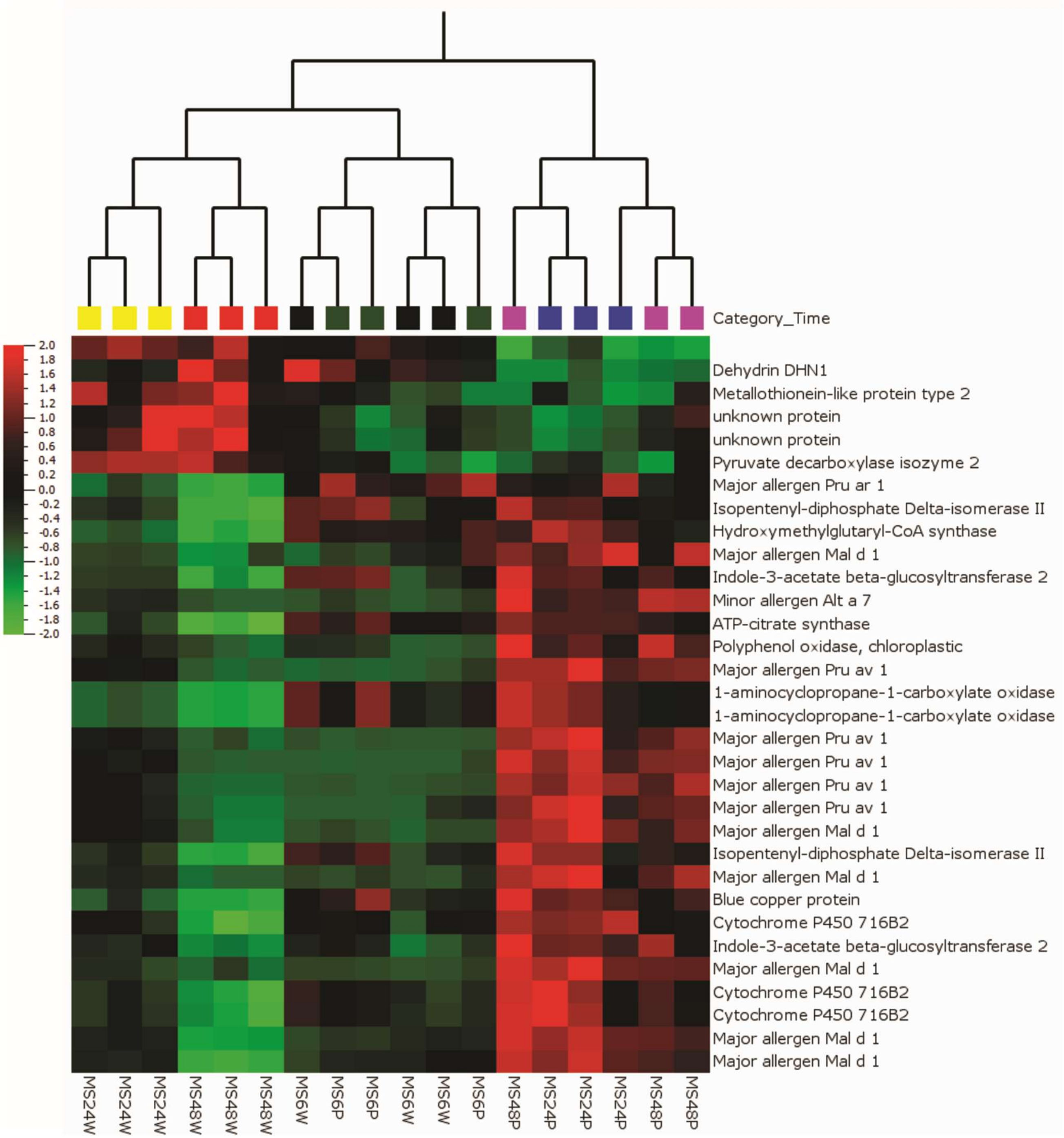

Supplementary Figure 3. Hierarchical clustering of differentially expressed genes in samples of *M. sieversii*-PI613981 (MS) apples at 6, 24, and 48 hours after wounding (W) or wounding/inoculation with *P. expansum* (P). Each lane represents an independent biological replicate ($p \leq 0.01$).

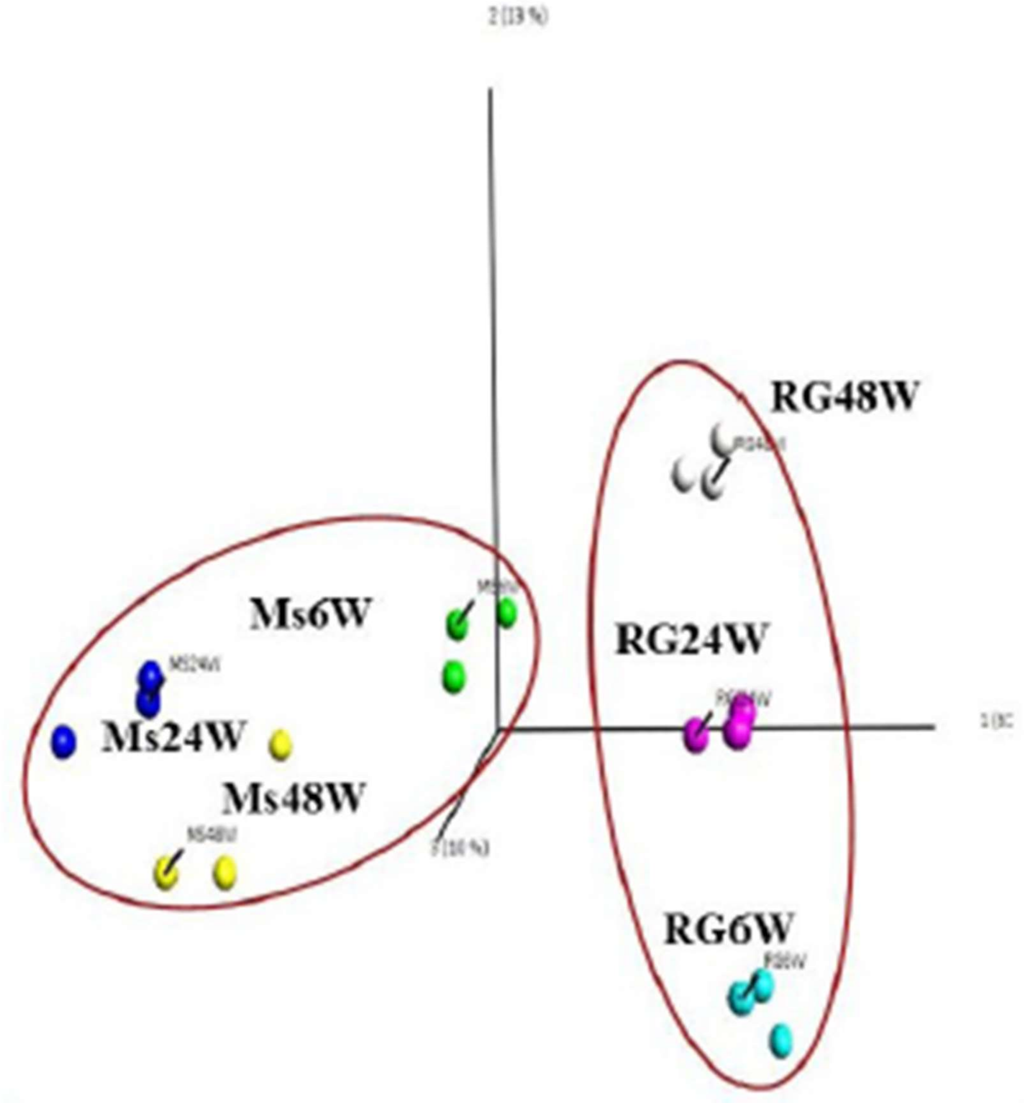

Supplementary Figure 4. Principal coordinates analysis (PCoA) of wounded *Malus sieversii*-PI613981 (denoted as MSW) and wounded 'Royal Gala' (denoted as RGW) apples at 6, 24 and 48 hours post treatment.

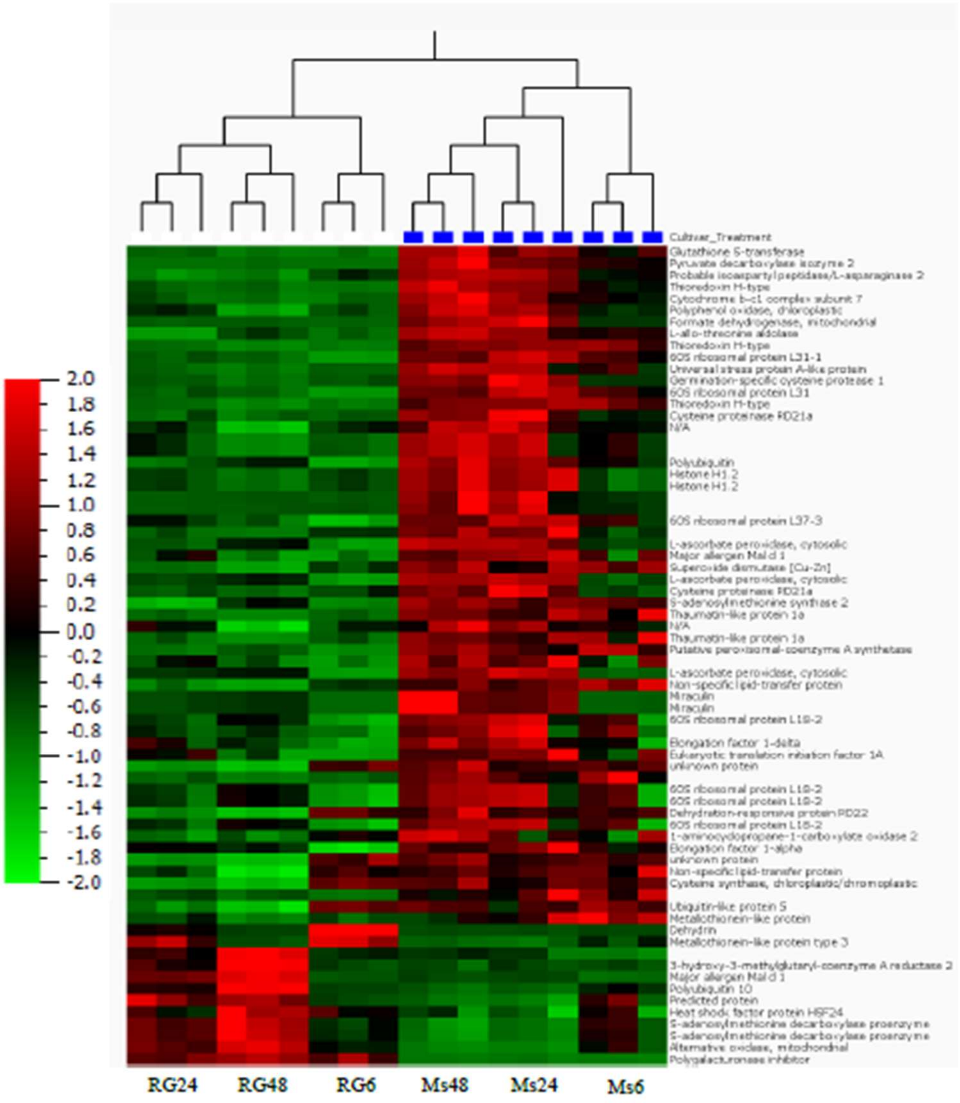

Supplementary Figure 5. Hierarchical clustering of differentially expressed genes in 'Royal Gala' (RG) *vs*. *M. sieversii*-PI613981 (MS) apples fruits at 6, 24, and 48 hours after wounding/inoculation with *P. expansum* (P). Each lane represents an independent biological replicate ($p \leq 0.01$).

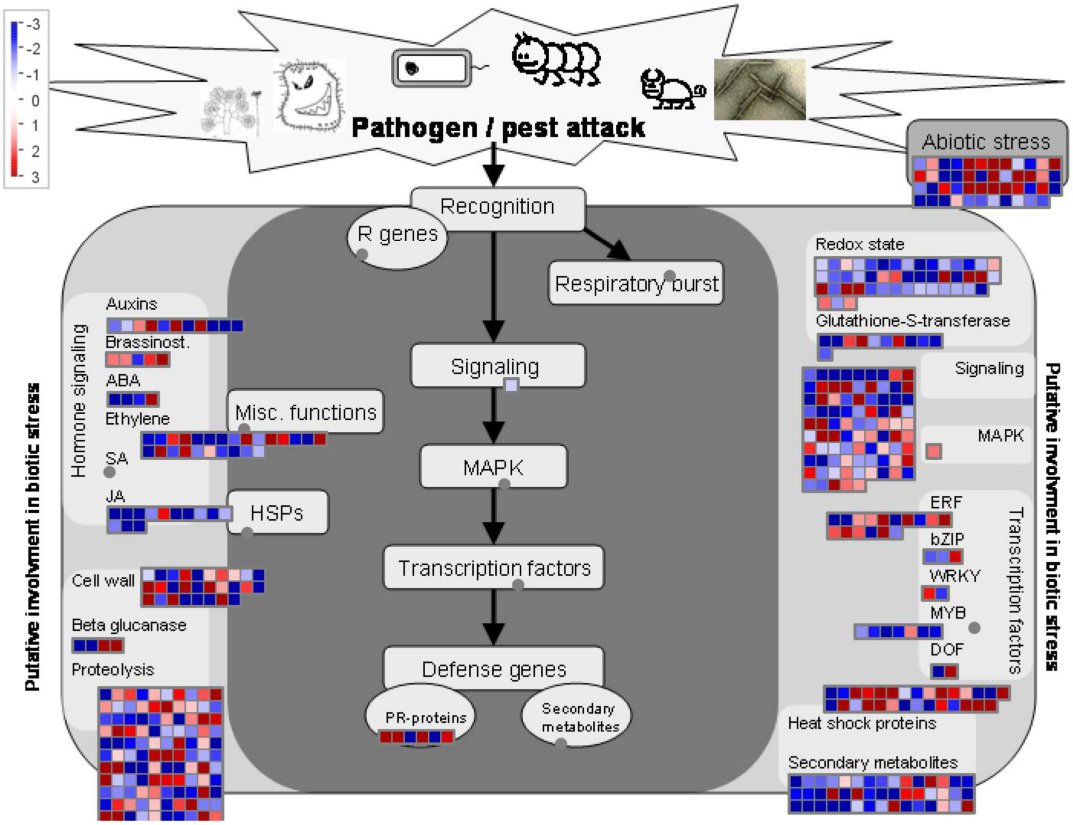

Supplementary Figure 6. Overview of biotic stress related MapMan bins for DEGs of both parental lines at time 0. Direct comparison of the two parental lines (all values given on a $\log_2$ scale). Genes with higher expression in the RG and in the MS parental lines are in red and blue, respectively. The scale bar displays changes in gene expression as $\log_2$ (ratio RG0/MS0) that were significant (FDR *p*-value$\leq$0.01 and $\log_2 \geq 1$ or ≤ -1) between both parental lines.

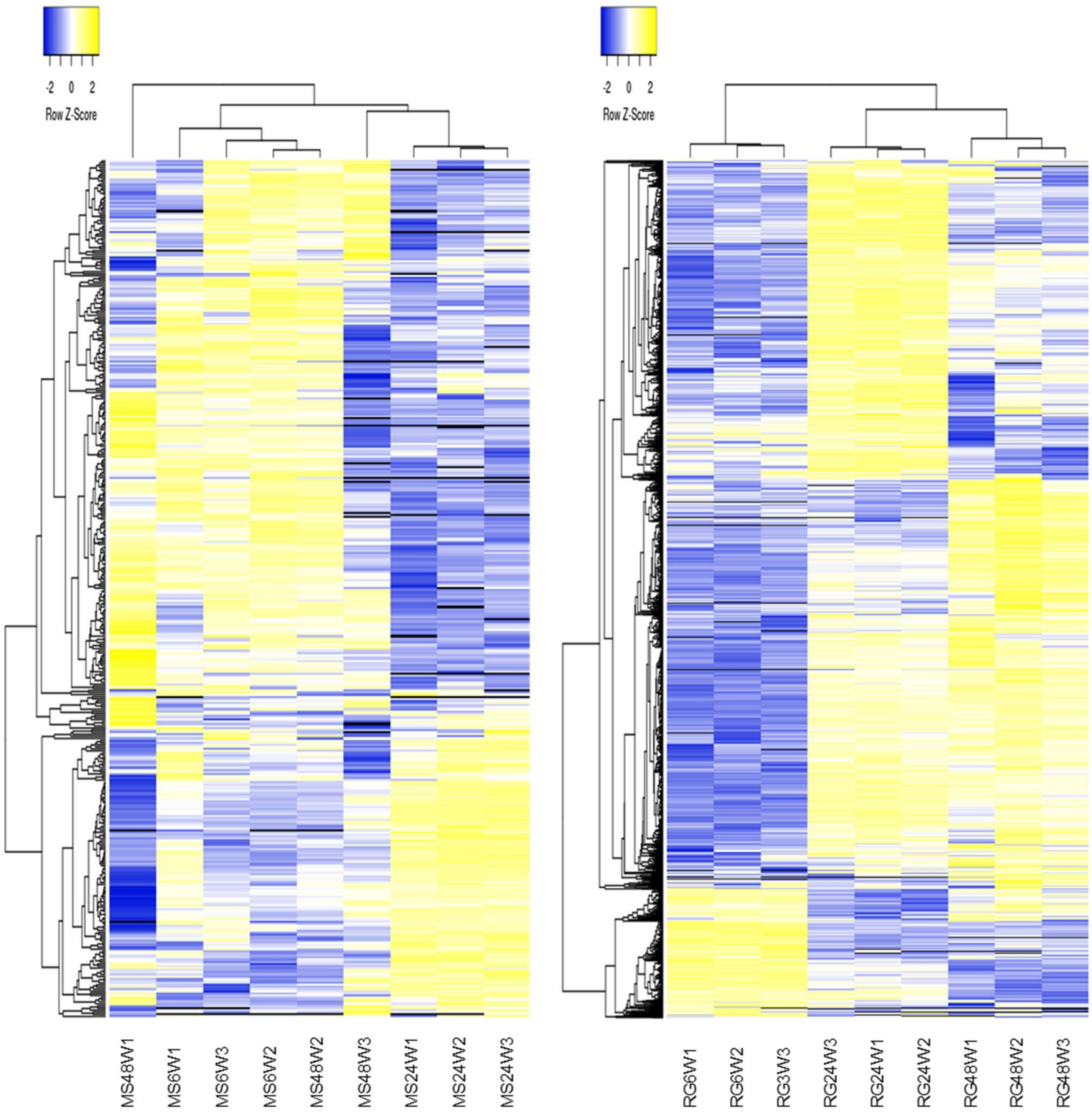

Supplementary Figure 7. Hierarchical cluster analysis and heatmap of differentially expressed genes in response to wounding within each parental line. The left dendogram corresponds to *Malus sieversii*-PI613981 (MS) apples and the right dendrogram corresponds to *Malus* × *domestica* 'Royal Gala' (RG) apples at 6, 24 and 48 hours after wounding. Data are expressed as $\log_2$ (ratio MS/MST0) or $\log_2$(RG/RGT0).

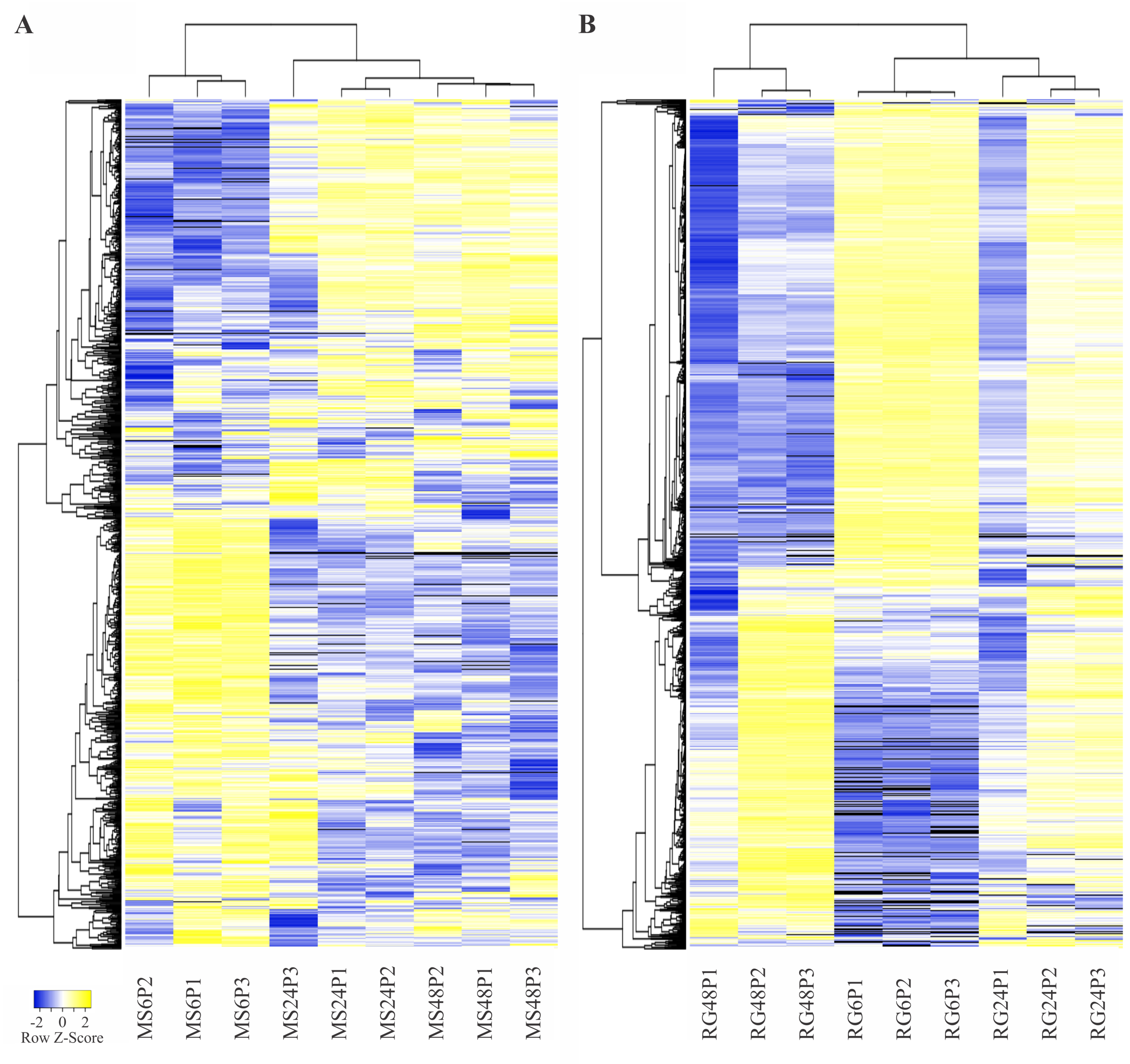

Supplementary Figure 8. Hierarchical cluster analysis and heatmap of differentially expressed genes in response to *P. expansum* inoculation within each parental line. The left dendogram corresponds to *Malus sieversii*-PI613981 (MS) apples and the right dendrogram corresponds to *Malus* × *domestica* 'Royal Gala' (RG) apples at 6, 24 and 48 hours after inoculation with *Penicillium expansum.* Data are expressed as $\log_2$ (ratio MS/MST0) or $\log_2$(RG/RGT0).

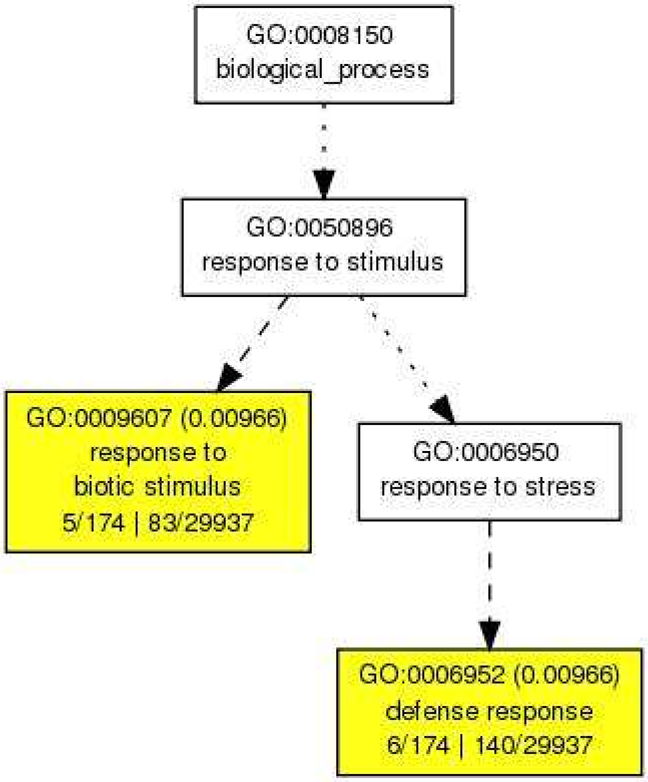

Supplementary Figure 9. Singular enrichment analysis (SEA) of significantly over-represented gene ontology (GO) terms in infected *Malus sieversii* fruit (ANOVA of $\log_2$(MS6P/MS0 vs $\log_2$(MS24P/MS0) vs $\log_2$(MS48P/MS0); FDR *p* value≤0.01) using AgriGO v2.0. Each box shows the GO term number, the *p*-value in parenthesis, and the GO term. The first pair of numerals represents the number of genes in the input list associated with that GO term and the number of genes in the input list. The second pair of numerals represents the number of genes associated with the particular GO term in the *Malus* x *domestica* database and the total number of apple genes with GO annotations in the *Malus* x *domestica* database. The box colors indicate levels of statistical significance with yellow ≤ 0.01.

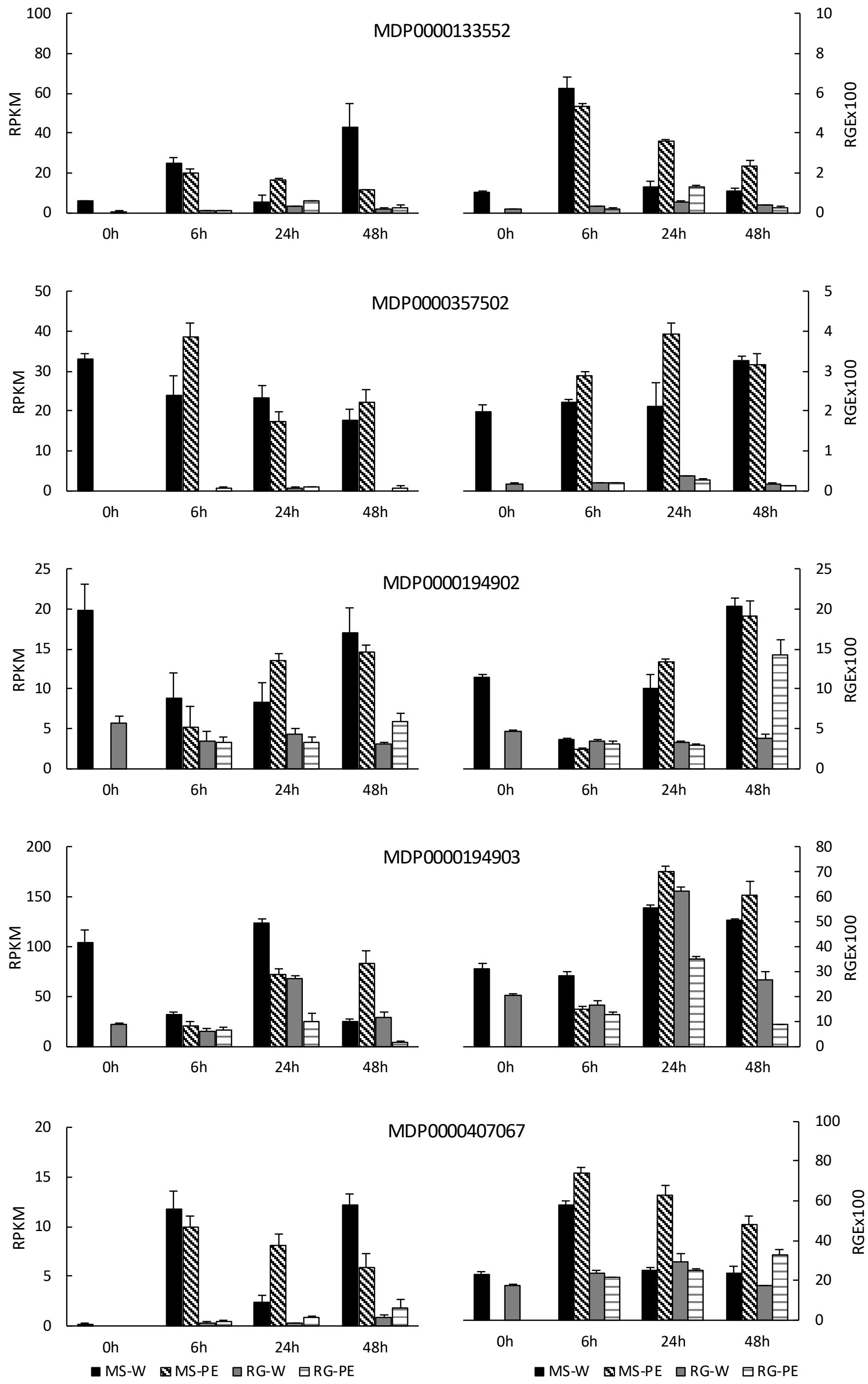

Supplementary Figure 10. Validation of RNA-Seq analysis by RT-qPCR. RPKM (reads per kilobase of exon per million fragments mapped) values obtained with RNA-Seq and relative gene expression (RGE) values obtained by RT-qPCR: wounded (W) or *P. expansum* (PE) infected in resistant *Malus sieversii*-PI613981 (MS) or susceptible 'Royal Gala' (RG) apples at 0, 6, 24 and 48 hpi. Error bars represent the standard error for three independent biological replicates.

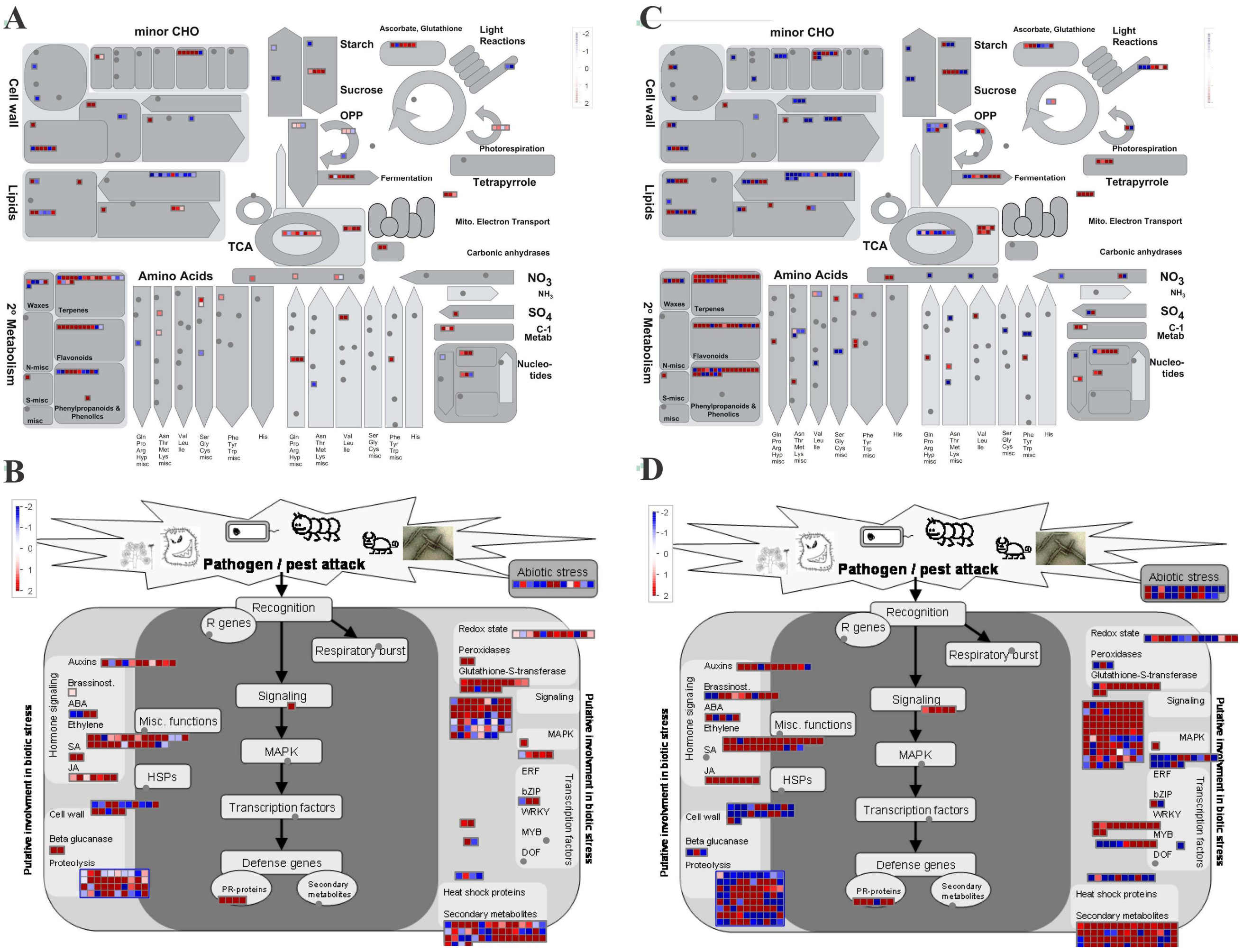

Supplementary Figure 11. Overview display of DEGs genes assigned to metabolic overview (A and C) and to biotic stress (B and D) at 24 hpi in *P. expansum* infected samples vs time 0 in the resistant MS parent (A and B) or in the susceptible RG parent (C and D). The scale bar displays changes in gene expression as the $\log_2$ ratio (MS24P/MS0) or $\log_2$ ratio (RG24P/RG0) that were significant (FDR *p* value≤0.01). Genes induced in response to *P. expansum* infection are highlighted in red and repressed genes are highlighted in blue. CHO: carbohydrates; OPP: oxidative pentose phosphate; TCA: tricarboxylic acid cycle.

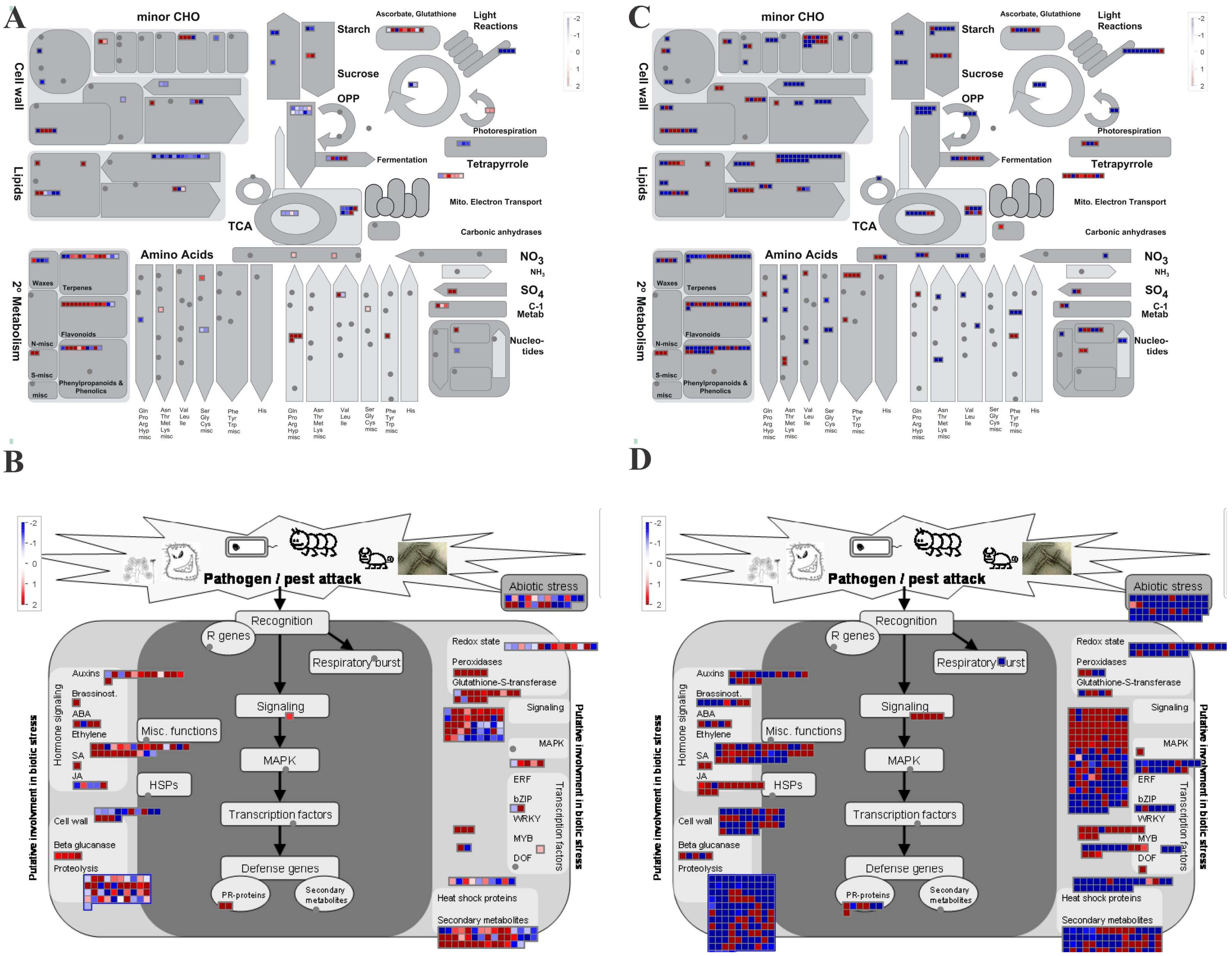

Supplementary Figure 12. Overview display of DEGs genes assigned to metabolic overview (A and C) and to biotic stress (B and D) at 48 hpi in *P. expansum* infected samples vs time 0 in the resistant MS parent (A and B) or in the susceptible RG parent (C and D). The scale bar displays changes in gene expression as the $\log_2$ ratio (MS48P/MS0) or $\log_2$ ratio (RG48P/RG0) that were significant (FDR *p* value≤0.01). Genes induced in response to *P. expansum* infection are highlighted in red and repressed genes are highlighted in blue. CHO: carbohydrates; OPP: oxidative pentose phosphate; TCA: tricarboxylic acid cycle.
